# Temperature–moisture–rainfall effects on internal dump slope stability in a cold-region open-pit coal mine with drainage validation

**DOI:** 10.1371/journal.pone.0352575

**Published:** 2026-09-21

**Authors:** Yu Wen, Ziling Song, Yifang Long, Zhenhua Yao, Shixiang Xiong, Wenhong Qian

**Affiliations:** 1 College of Mining, Liaoning Technical University, Fuxin, Liaoning, China; 2 College of Environmental Science and Engineering, Liaoning Technical University, Fuxin, Liaoning, China; 3 School of Mechanics and Engineering, Liaoning Technical University, Fuxin, Liaoning, China; China University of Mining and Technology, CHINA

## Abstract

Internal dump slopes in cold-region open-pit coal mines are subjected to moisture variation, freezing temperatures, rainfall infiltration, and groundwater seepage. This study developed an integrated experimental-numerical-field framework to evaluate these effects for the Chaoyang open-pit coal mine in Heilongjiang Province, China. Unconfined compression and direct shear tests were conducted under four moisture contents of 14.0%, 17.6%, 23.0%, and 26.0% and three temperatures of 23 ± 2°C, −5°C, and −15°C. The measured elastic modulus, cohesion, and internal friction angle were incorporated into a three-dimensional GTS NX model to assess slope responses before and after 72 h of rainfall.At room temperature, increasing moisture content reduced the elastic modulus from 1802.4 to 1003.6 MPa and cohesion from 18 to 10 kPa, while the pre-rainfall factor of safety decreased from 1.405 to 1.176. Under subzero conditions, stiffness and cohesion increased because of pore-ice bonding, whereas the internal friction angle varied only modestly and non-monotonically. At −15°C, the post-rainfall factor of safety ranged from 1.478 to 1.537, and rainfall-induced reductions under all subzero conditions remained below 0.60%. Under the room-temperature natural-moisture condition, drainage pipes increased the factors of safety before and after rainfall from 1.221 and 1.184 to 1.413 and 1.384, respectively. Field monitoring recorded an average drainage discharge of 2.51 × 10^4^ m^3^/day and groundwater-level drawdowns of 12.19 and 9.94 m in the upper and middle aquifers.The results demonstrate that moisture weakens unfrozen dump materials but can enhance frozen-state strength through ice bonding. The integration of laboratory-derived temperature-moisture parameters, three-dimensional rainfall analysis, and field hydraulic validation provides a practical framework for stability assessment and drainage control of cold-region internal dump slopes.

## 1. Introduction

Internal dumping is widely used in large open-pit coal mines to dispose of stripped overburden materials within mined-out areas. Unlike natural slopes, internal dump slopes are artificial accumulation bodies composed of reconstituted and heterogeneous materials and are generally characterized by loose structure, uneven particle-size distribution, variable compaction, well-developed pores, weak interparticle bonding, and complex hydrogeological conditions. Their stability is therefore sensitive to slope geometry, material strength degradation, groundwater seepage, rainfall infiltration, mining disturbance, and climatic forcing. The overall slope angle is an important geometric factor controlling open-pit slope stability, and Sanei et al. recently investigated its influence on the stability of an open-pit mine slope [1]. Physical and numerical modelling methods have also become important approaches for identifying displacement evolution, strain localization, crack development, and potential failure zones in mining slopes [2,3]. For internal dump slopes, these problems are particularly prominent because the dumped materials are reconstituted, weakly cemented, and strongly affected by groundwater and environmental changes.

The pronounced heterogeneity and discontinuity of mine-dump materials have prompted the application of numerical methods capable of representing spatial variability and dynamic disturbance. Mohanty et al. investigated the response of a coal-mine overburden dump slope to blast-induced vibration using a discrete element method [4]. In another study, an extended finite-element method combined with a Voronoi tessellation scheme was applied to assess the seismic performance of a coal-mine overburden dump slope while representing the spatial heterogeneity of the dumped material [5]. These studies demonstrate the relevance of discontinuum and heterogeneous numerical approaches for analyzing dump slopes subjected to dynamic loading. However, their primary focus was blasting- or earthquake-induced behavior, whereas the coupled effects of moisture content, freezing temperature, rainfall infiltration, and drainage on cold-region internal dump slopes remain insufficiently understood. In addition, particle- or block-based models generally require extensive mesoscopic parameter calibration and considerable computational resources when applied to large three-dimensional slopes. For global seepage, deformation, and stability assessment, continuum finite-element models remain applicable when laboratory-derived parameters are interpreted as equivalent mechanical properties of heterogeneous dump materials.

Water is one of the most important factors affecting the stability of open-pit mine slopes and internal dump slopes. In unsaturated geomaterials, matric suction contributes to apparent cohesion and shear strength. The classical shear-strength framework for unsaturated soils proposed by Fredlund et al.[6]provides a theoretical basis for describing the contribution of matric suction to soil strength. Rainfall infiltration and groundwater recharge can alter the hydraulic state of slope materials, increase moisture content and pore water pressure, reduce matric suction and effective stress, and consequently decrease shear strength. For slopes in multi-aquifer open-pit mines, groundwater flow fields and hydrostatic-pressure distributions may significantly influence slope stability, particularly where aquifers occur within or are exposed in the slope body [7]. Numerical studies on unsaturated slopes have further shown that rainfall-induced instability depends strongly on hydraulic boundary conditions, initial moisture state, crack development, infiltration pathways, and drainage conditions [8,9]. These findings indicate that pore water pressure redistribution, saturation evolution, and material-strength degradation should be considered together when evaluating internal dump slope stability under rainfall conditions.

In cold-region open-pit coal mines, the role of water becomes more complex because temperature controls the phase state of pore water. Under unfrozen conditions, increasing moisture content generally reduces matric suction, weakens direct particle contacts, promotes lubrication and particle rearrangement, and decreases material stiffness and shear strength. Under subzero temperatures, however, part of the pore water freezes and forms pore ice and ice bridges between particles, while a certain amount remains as unfrozen water around particle surfaces and within fine pores. The resulting soil–ice structure can enhance apparent cohesion, restrict particle movement, and increase stiffness and shear resistance. Previous reviews and experimental studies have shown that the mechanical behavior of frozen geomaterials is governed by the coexistence of soil particles, pore ice, and unfrozen water and is strongly affected by temperature and water–ice content [10]. Direct shear tests on frozen coarse-grained soils have also demonstrated that temperature-induced strength variation is commonly reflected more strongly in cohesion than in internal friction angle [11]. Similar ice-bonding effects have been observed in frozen granular materials, where moisture may influence mechanical strength in a nonlinear manner under subzero conditions [12]. Therefore, moisture can act mainly as a weakening factor under unfrozen conditions but may contribute to mechanical strengthening when sufficient pore water freezes.

Despite these advances, several connected issues remain unresolved. First, studies considering the heterogeneity of mine-dump materials through discrete-element or Voronoi-based approaches have mainly focused on particle-scale deformation, blast loading, or earthquake loading. They do not directly clarify how moisture content, freezing temperature, rainfall infiltration, and groundwater conditions jointly affect cold-region internal dump slopes. Second, frozen-soil studies have largely concentrated on specimen-scale mechanical behavior, whereas rainfall and groundwater models commonly use fixed or independently selected mechanical parameters. The transfer of experimentally determined temperature–moisture-dependent properties into slope-scale seepage–deformation–stability analysis remains limited. Third, internal dump slopes differ from natural slopes, external dumps, and final pit slopes because they consist of reconstituted heterogeneous materials and may contain multiple aquifers and aquitards. The combined effects of material state, rainfall infiltration, groundwater redistribution, and drainage layout in such slopes have not been sufficiently quantified. Finally, although horizontal drains and perforated drainage pipes are widely used to lower groundwater levels and pore water pressure [13–15], few studies have combined three-dimensional drainage analysis with field measurements of drainage discharge and aquifer drawdown for a cold-region internal dump slope.

To address these gaps, the north slope of the internal dump at the Chaoyang open-pit coal mine in Heilongjiang Province, China, was selected as the study object. The investigation followed a four-stage workflow. First, field engineering geological and hydrogeological data were used to characterize the slope geometry, aquifer–aquitard structure, groundwater conditions, and representative dump material. Second, unconfined compression and direct shear tests were conducted under four moisture contents—14.0%, the field-measured natural moisture content of 17.6%, 23.0%, and 26.0%—and three temperature conditions of 23 ± 2°C, −5°C, and −15°C. The elastic modulus, cohesion, and internal friction angle were independently determined for the resulting 12 prescribed temperature–moisture states.

Third, the laboratory-derived mechanical parameters were assigned to the corresponding internal-dump material domains in a three-dimensional GTS NX finite-element model. The model was used to compare pore water pressure, saturation, displacement, shear-strain distribution, and factor of safety before and after a prescribed rainfall infiltration event of 0.05 m^3^/(m^2^·day) lasting 72 h. Fourth, HDPE perforated drainage pipes were introduced into the upper and middle aquifers to evaluate drainage-induced changes in the hydraulic and stability responses of the slope. Field monitoring of drainage discharge and groundwater-level drawdown was subsequently used to assess the hydraulic effectiveness of the drainage system.

The objectives of this study are to: (1) quantify the effects of moisture content and temperature on the stiffness and shear strength of internal-dump materials; (2) establish the relationship between laboratory-determined mechanical parameters and slope-scale numerical assessment; (3) determine how prescribed temperature–moisture states influence rainfall-induced seepage, deformation, and factor-of-safety changes; and (4) evaluate the hydraulic and stabilizing effects of HDPE perforated drainage pipes through numerical analysis and field monitoring. The principal contribution of this study is the integration of temperature–moisture-dependent laboratory measurements, three-dimensional rainfall seepage–deformation–stability analysis, and field drainage validation within a unified framework. The study represents a prescribed-state comparative assessment rather than a transient thermo–hydro–mechanical simulation of the complete freezing and thawing process.

## 2. Study area and materials

### 2.1. Study area

**Fig 1:**
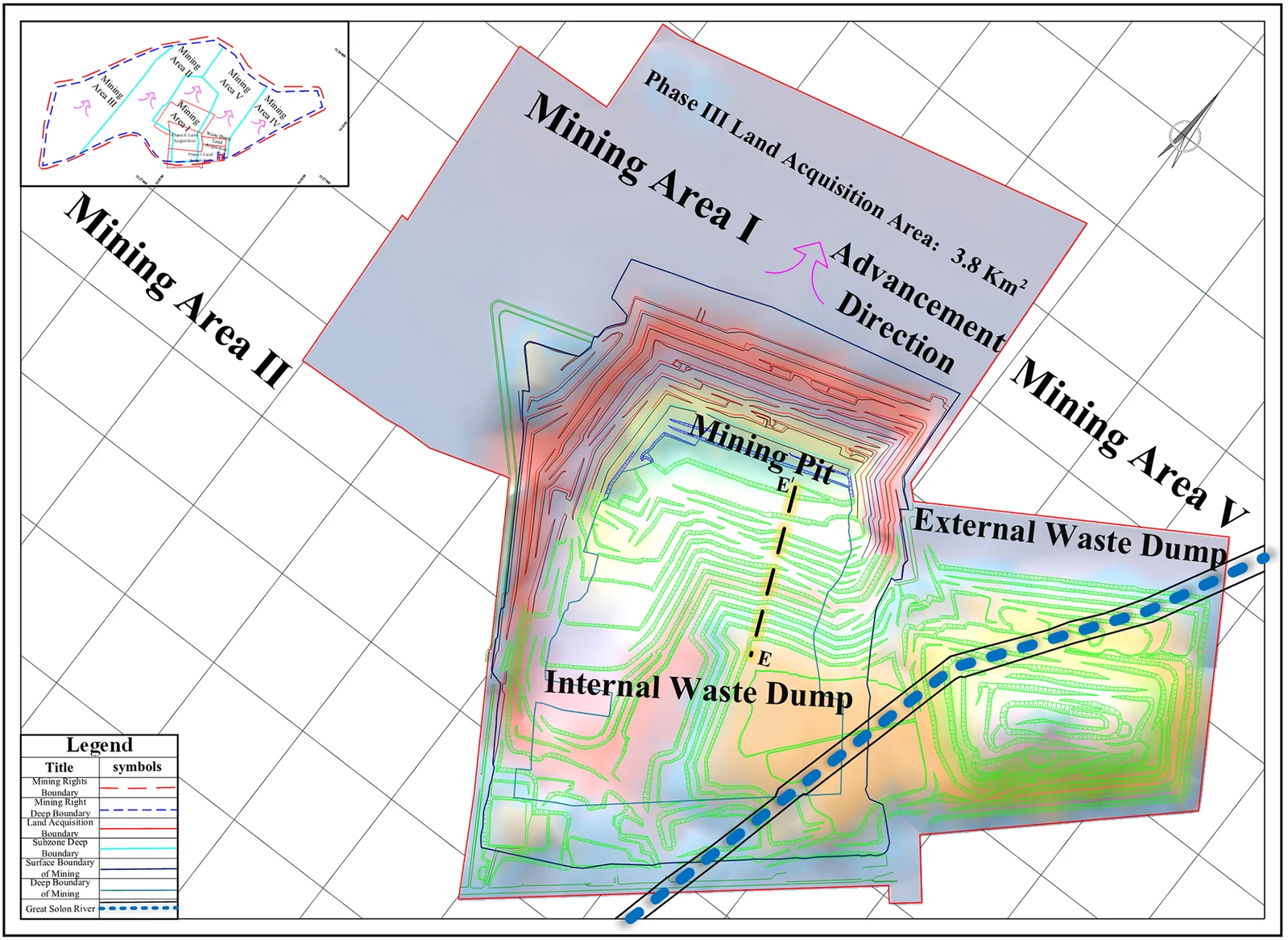
Current engineering layout of the Chaoyang open-pit coal mine and its internal dump. Adapted by the authors from the original DWG/CAD engineering layout drawing provided by Long Zhang, under a CC BY 4.0 license, with permission from Long Zhang, original copyright 2026.

The Chaoyang open-pit coal mine is located in Baoqing County, Shuangyashan City, Heilongjiang Province, China, and is situated in a cold region of Northeast China. The study area is characterized by long and cold winters and pronounced seasonal freezing. The slope geomaterials are therefore subjected to multiple environmental factors, including low-temperature freezing, freeze–thaw transition, rainfall infiltration, and groundwater seepage. During mining, a large amount of stripped overburden material is dumped into the mined-out pit, forming artificial accumulation slopes in the internal dump, as shown in Fig 1. Owing to the complex sources of dumped materials, uneven particle-size distribution, and variable compaction states, the internal dump slope exhibits strong heterogeneity and hydro-mechanical sensitivity [16].

**Fig 2:**
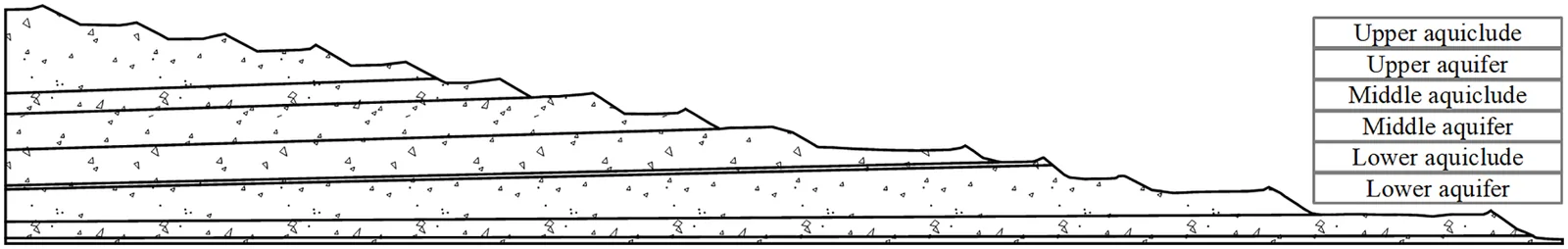
Representative E–E′ section of the north slope of the internal dump in the Chaoyang open-pit coal mine.

In this study, the north slope of the internal dump in the Chaoyang open-pit coal mine was selected as the research object, and the representative E–E′ section was used as the basis for laboratory parameter determination and numerical simulation analysis, as shown in Fig 2. This area is strongly affected by groundwater seepage and rainfall infiltration, and can represent typical stability problems of internal dump slopes in cold-region open-pit coal mines under hydrothermal coupling conditions [17].

### 2.2. Hydrogeological conditions

**Fig 3:**
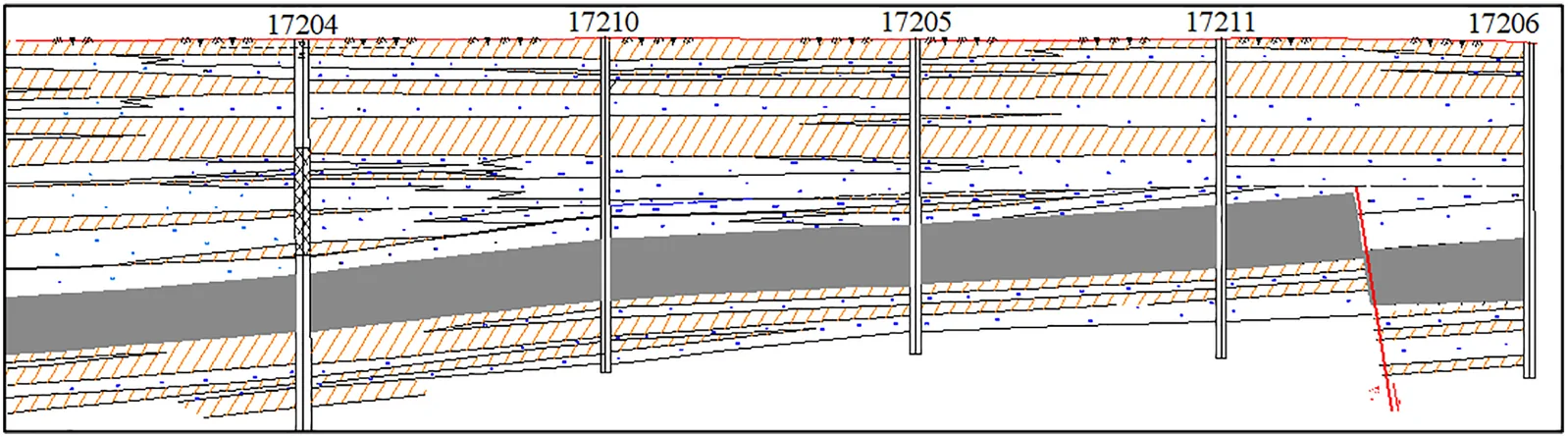
Hydrogeological structure of the internal dump slope.

The hydrogeological structure of the internal dump slope is relatively complex and mainly consists of alternating aquitards and aquifers. According to field engineering geological and hydrogeological data, the slope can be divided from top to bottom into the upper aquitard, upper aquifer, middle aquitard, middle aquifer, lower aquitard, and lower aquifer, as shown in Fig 3. This multilayer hydrogeological structure controls groundwater occurrence, pore water pressure distribution, and water migration pathways after rainfall infiltration, making it an important factor affecting slope stability.

The upper aquitard is continuously distributed in the study area and directly exposed at the ground surface. This layer is characterized by relatively fine-grained material, soft-plastic behavior, and increased hardness after drying. Its average thickness is approximately 7.15 m, with a maximum thickness of 21.55 m. The layer is relatively thicker in the northeastern part of the area, where the local thickness exceeds 10 m, and it generally exhibits good water-blocking capacity.

The upper aquifer has an average thickness of approximately 11.75 m, with a unit water inflow of 0.336 L/(s·m) and a permeability coefficient of 10.447 m/d. The groundwater in this aquifer is pore water and occurs as confined water. The overall groundwater flow direction is from south to north, with a hydraulic gradient of approximately 1.4%. The groundwater type is HCO_3_–Cl–Ca–Na, with a mineralization of approximately 206.04 mg/L, a pH value of approximately 6.5, and a water temperature of approximately 5°C.

The middle aquitard occurs between the upper and middle aquifers and is relatively stable throughout the study area. This layer is dense and plastic, with an average thickness of approximately 7.59 m, a maximum thickness of 19.10 m, and a minimum thickness of approximately 1.55 m. In general, the layer is thicker in the central area and gradually becomes thinner toward the surrounding areas. In the western part, it locally develops into an interbedded structure.

The middle aquifer has an average thickness of approximately 21.9 m. It is generally thinner in the southern part and thicker in the northern part, and it becomes further thinner in the southeastern part due to sedimentary conditions. The unit water inflow is 0.298 L/(s·m), and the permeability coefficient is 1.344 m/d. The groundwater in this aquifer is also confined pore water, with a water type of HCO_3_–Cl–Na–Ca. The overall groundwater flow direction is from south to north. Owing to its large thickness, this aquifer is an important layer controlling pore water pressure and groundwater discharge conditions within the slope.

The lower aquitard is located between the coal seam floor and the lower aquifer. Its thickness varies considerably in space, and its development is relatively unstable, with local absence in some areas. The thickness ranges from approximately 0 to 13.55 m, with an average thickness of approximately 3.40 m. Areas with greater thickness show relatively good water-blocking capacity, whereas locally absent or thinner zones may become potential pathways for vertical groundwater connection.

The lower aquifer has an average thickness of approximately 6.57 m and poor continuity, with local absence in some areas. It is relatively thicker in the southern part and thinner in the northern part. The unit water inflow is 0.318 L/(s·m), and the permeability coefficient is 6.629 m/d. The groundwater type is HCO_3_–SO_4_–Na–Ca, with a mineralization of approximately 190.015 mg/L, a pH value of approximately 6.5, and a water temperature of approximately 6°C. Although this aquifer is relatively thin, it may influence the hydraulic conditions at the lower part of the slope in areas where the aquitard is locally absent.

Overall, the internal dump slope of the Chaoyang open-pit coal mine has a distinct multilayer aquifer–aquitard structure. The upper and middle aquifers are the key layers controlling groundwater occurrence and pore water pressure distribution within the slope, and they are also the main targets for drainage pipe installation and groundwater control. Therefore, the stability assessment of this slope should consider aquifer distribution, rainfall infiltration, pore water pressure variation, and the influence of drainage measures on the hydraulic state of the slope.

### 2.3. Sampling and material characteristics

The test material was collected from a representative location of the north slope of the internal dump in the Chaoyang open-pit coal mine and can represent the basic engineering properties of the internal-dump material in the study area. The material is mainly composed of rock–soil mixtures stripped and dumped during open-pit mining. It is characterized by complex particle composition, loose structure, well-developed pores, and strong heterogeneity. Because the internal-dump material has experienced dumping, accumulation, and local compaction, its mechanical properties differ significantly from those of natural undisturbed geomaterials.

After sampling, the internal-dump material was air-dried, crushed, sieved, and weighed for specimen preparation. Four gravimetric moisture contents were selected to represent a progressive range of field-relevant moisture states. The natural moisture content measured from the field sample was 17.6%, which was used as the baseline moisture condition. A moisture content of 14% represented a relatively low-moisture state below the natural condition, whereas 23% and 26% represented medium-to-high and high-moisture states above the natural condition, respectively. The four moisture levels therefore enabled comparison of the mechanical response of the internal-dump material over a range extending from relatively dry to high-moisture conditions.

Three temperature conditions were considered. Room temperature (RT) represented the unfrozen state, while −5°C and −15°C represented moderate and stronger freezing conditions, respectively. The resulting four moisture contents and three temperatures formed 12 temperature-moisture combinations for the laboratory tests.

## 3. Methods

### 3.1. Experimental design

To obtain the key mechanical parameters of the rock–soil mixture from the internal dump of the Chaoyang open-pit coal mine under different hydrothermal conditions, laboratory mechanical tests were designed under various combinations of moisture content and temperature. Four moisture-content levels were considered: 14%, natural moisture content of 17.6%, 23%, and 26%. The 14% moisture content represents a relatively low-moisture condition; the natural moisture content of 17.6% represents the field-measured initial moisture state; and the 23% and 26% moisture contents represent medium-to-high and high-moisture conditions, respectively. Three temperature levels were selected: RT, −5°C, and −15°C, corresponding to the unfrozen state, moderate freezing state, and stronger freezing state, respectively. Accordingly, 12 temperature–moisture combinations were established.

For the 12 working conditions, unconfined compression tests and direct shear tests were conducted. The unconfined compression tests were used to obtain the stress–strain curves and elastic modulus of the specimens under different temperature and moisture-content conditions. The direct shear tests were used to obtain the shear stress–shear displacement curves, and the cohesion and internal friction angle were determined by fitting the shear strength envelopes. The obtained elastic modulus, cohesion, and internal friction angle were further used as key mechanical input parameters in the subsequent GTS NX numerical simulations, ensuring that the numerical model could reflect the mechanical characteristics of the internal-dump rock–soil mixture under different environmental conditions.

For the unconfined compression tests, three independent specimens were prepared and tested for each of the 12 temperature-moisture combinations, resulting in a total of 36 unconfined compression specimens. The elastic modulus reported for each condition was calculated as the arithmetic mean of the three independently measured values.

For the direct shear tests, three specimens were prepared for each temperature-moisture combination, with one specimen tested at each normal stress of 50, 100, and 200 kPa. A total of 36 direct shear specimens were therefore tested. The three specimens within each temperature-moisture group represented three different normal-stress levels rather than replicate tests under the same normal stress. The peak shear stresses obtained at the three normal stresses were linearly fitted to construct the Mohr-Coulomb strength envelope, from which cohesion and internal friction angle were determined.

This testing scheme provided repeated measurements for the unconfined compression response and sufficient normal-stress levels for determination of the direct shear strength parameters.

### 3.2. Specimen preparation and moisture conditioning

The rock-soil mixture used in the tests was collected from a representative location on the north slope of the internal dump. After sampling, the material was air-dried and manually crushed, and oversized rock fragments and visible impurities were removed. The material was then sieved and thoroughly mixed to obtain a comparatively uniform particle composition for specimen preparation.

The target dry unit weight of the remolded specimens was 18.522 kN/m^3^, corresponding to a dry density of 1.889 g/cm^3^. The moisture content of the specimens was controlled on a gravimetric basis according to ASTM D2216 [18]. For each target moisture condition, the required amount of water was calculated from the initial dry mass of the material and was gradually added by spraying during continuous mixing. The moisture-conditioned material was sealed for 48 h to promote uniform redistribution of water among the particles.

The specimens were prepared using a consistent layered filling and compaction procedure. The filling mass and compaction procedure were maintained consistently among specimens to minimize preparation-induced differences. Before testing, the actual moisture content was verified gravimetrically and controlled within ±1 percentage point of the target value. Specimens exceeding this allowable deviation were not used directly; the material was remixed, moisture-adjusted, and prepared again. After molding, the specimens were labeled and sealed with plastic film to minimize moisture loss before temperature conditioning and mechanical testing.

In this study, RT was maintained at 23 ± 2°C during specimen conditioning and mechanical testing. Specimens assigned to the −5°C and −15°C conditions were sealed with plastic film and placed in a low-temperature chamber at the prescribed temperature for 12 h before testing. The same conditioning duration was applied to all specimens within the corresponding subzero-temperature groups. After conditioning, each specimen was rapidly transferred from the chamber to the testing apparatus to minimize temperature change and moisture loss before loading.

**Fig 4:**
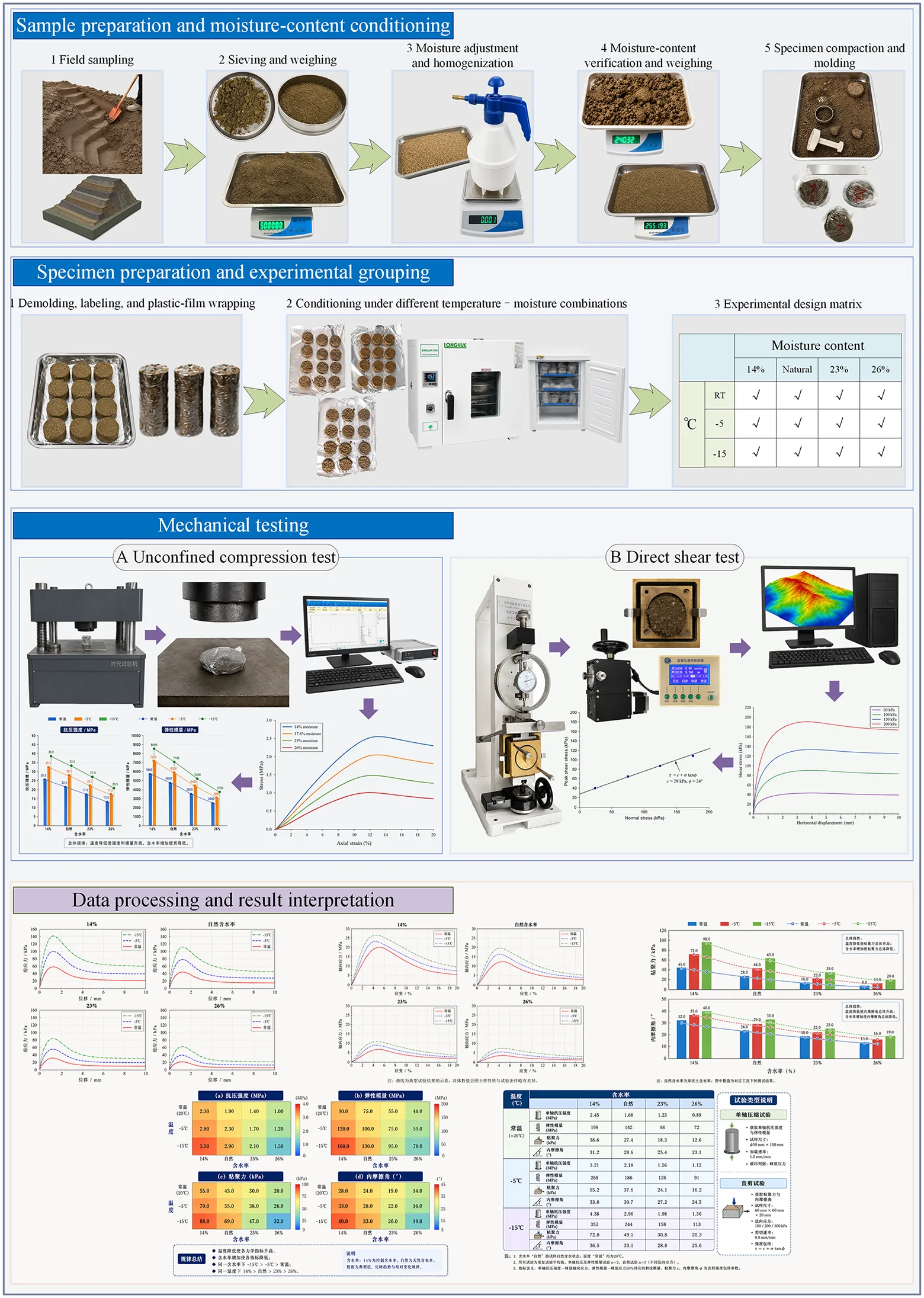
Workflow of specimen preparation, mechanical testing, and data analysis under different temperature–moisture conditions. All photographs, plots, screenshots, and diagrams in this figure were created by the authors.

Because the internal specimen temperature was not continuously monitored during conditioning, the subzero-temperature conditions were defined by the chamber temperature and the consistent 12 h conditioning period. Therefore, the specimens were considered to have approached the prescribed temperature state rather than being directly verified to have reached a completely uniform internal temperature. The specimen preparation and testing procedure is shown in Fig 4.

### 3.3. Unconfined compression test

The unconfined compression test was conducted with reference to ASTM D2166/D2166M-24, which is used to determine the unconfined compressive strength and deformation response of remolded or reconstituted soil specimens [19]. A total of 12 temperature–moisture combinations were considered, including four moisture contents, namely 14%, natural moisture content of 17.6%, 23%, and 26%, and three temperature levels, namely RT, −5°C, and −15°C. Replicate specimens were prepared for each condition to obtain representative unconfined compressive stress–strain curves and elastic modulus values.

The unconfined compression specimens had dimensions of 61.8 mm × 80 mm, with a diameter of 61.8 mm and a height of 80 mm. Before testing, the moisture-conditioned and molded specimens were conditioned at the target temperatures. The room-temperature specimens were tested under indoor laboratory conditions, whereas the specimens for the −5°C and −15°C conditions were conditioned in a low-temperature chamber for 12 h to ensure that the internal temperature of the specimens approximately reached the prescribed state. Before testing, the specimens were quickly removed and placed at the center of the loading platen, ensuring full contact between the upper and lower specimen surfaces and the loading platens to reduce the influence of eccentric loading.

During the test, axial load and axial deformation were recorded simultaneously, and axial stress and axial strain were calculated based on the initial cross-sectional area and height of the specimen. Loading was continued until obvious specimen failure occurred or the axial stress decreased markedly after reaching the peak value. The obtained unconfined compressive stress–strain curves were used to characterize the compressive deformation response of the specimens under different temperature and moisture-content conditions. The elastic modulus was calculated from the approximately linear portion of the stress–strain curve, and the mean value of replicate specimens was used for each working condition. The obtained elastic modulus values were then used as important deformation parameters for the subsequent GTS NX numerical simulations.

### 3.4. Direct shear test

The direct shear test was conducted with reference to ASTM D3080/D3080M-23, which is applicable for determining the drained shear strength of soils under different normal stresses and for deriving the Mohr strength envelope and shear strength parameters [20]. 12 temperature–moisture combinations were considered, including four moisture contents of 14%, natural moisture content of 17.6%, 23%, and 26%, and three temperature levels of RT, −5°C, and −15°C. The shear stress–shear displacement curves were obtained for each condition, and the cohesion and internal friction angle were further determined.

The direct shear specimens had dimensions of 61.8 mm × 20 mm, with a diameter of 61.8 mm and a height of 20 mm. Before testing, the moisture-conditioned specimens were conditioned at the target temperatures. The room-temperature specimens were tested under indoor laboratory conditions, whereas the specimens for the −5°C and −15°C conditions were conditioned in a low-temperature chamber for 12 h to ensure that the specimens reached the target temperature state. After being placed in the direct shear box, the specimens were subjected to three normal stress levels of 50 kPa, 100 kPa, and 200 kPa, and shearing was performed after the normal stress stabilized.

**Fig 5:**
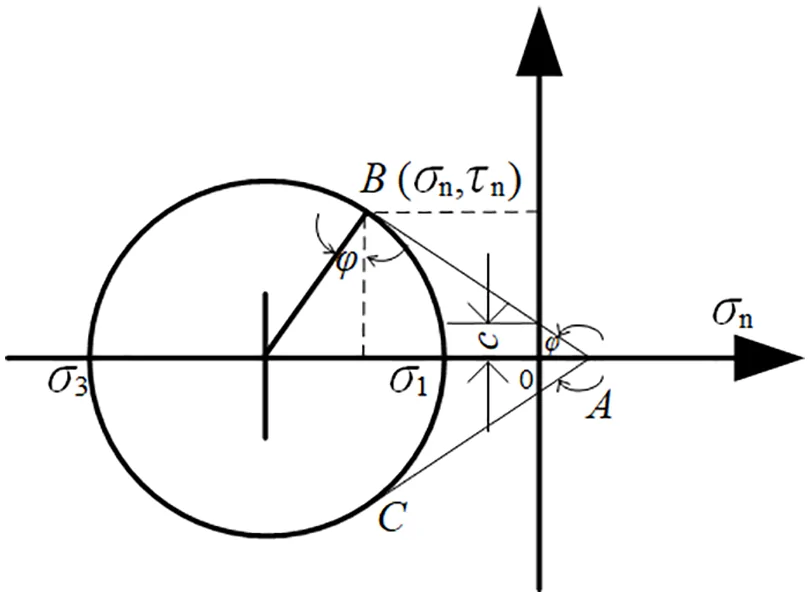
Fitting method for shear strength envelopes of internal-dump rock–soil mixture specimens under different temperature and moisture-content conditions.

The direct shear test was performed at a constant shear rate of 0.8 mm/min. During the test, shear force and shear displacement were continuously recorded, and shear stress was calculated based on the shear area. The shearing process was continued until the shear stress reached a peak and tended to stabilize, or until the specified shear displacement was reached. The peak shear stresses obtained under different normal stresses were used to fit the Mohr-Coulomb strength envelope, from which the cohesion and internal friction angle under each temperature-moisture condition were determined, as shown in Fig 5.

According to the Mohr-Coulomb strength criterion, the shear strength can be expressed as:


$$\begin{array}{c} \tau_{n}=C+\sigma_{n}tan\varphi \end{array}$$ (1)

where *τ* is the peak shear stress, *c* is cohesion, *σ*_n_ is the normal stress, and φ is the internal friction angle. By linearly fitting the peak shear stresses under normal stresses of 50 kPa, 100 kPa, and 200 kPa, cohesion *c* was obtained from the intercept, and the internal friction angle *φ* was calculated from the slope. The obtained cohesion and internal friction angle were used to characterize the shear strength of the specimens under different temperature and moisture-content conditions and served as key strength parameters in the subsequent numerical simulations of slope stability.

### 3.5. Numerical modelling in GTS NX

**Fig 6:**
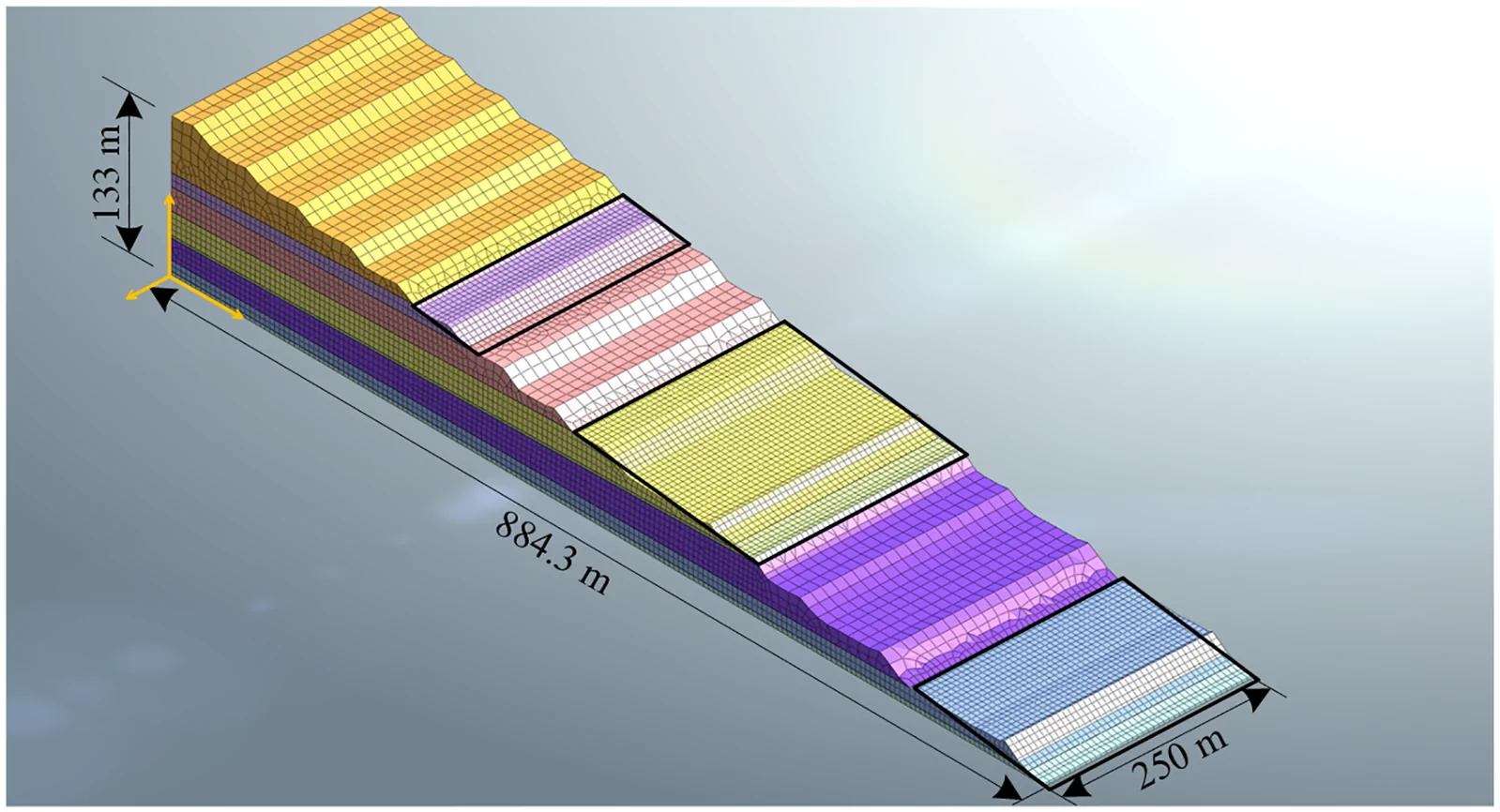
Finite element model of the internal dump slope in GTS NX and locally refined mesh regions.

To further analyze the seepage–deformation–stability response of the internal dump slope under different temperature, moisture-content, and rainfall conditions, a numerical model of the north slope of the internal dump in the Chaoyang open-pit coal mine was established using the geotechnical finite element software GTS NX. Based on field surveying and engineering geological investigation data, the representative E-E′ section, which is strongly affected by groundwater seepage, was selected for modelling. The model dimensions were 884.3 m in length, 133 m in height, and 250 m in transverse width, which allowed the slope geometry, stratigraphic structure, and aquifer distribution of the study area to be reasonably represented, as shown in Fig 6.

The Mohr-Coulomb elastoplastic model was adopted to describe the mechanical response of the internal-dump rock-soil mixture. This model was selected because the direct shear tests provided the cohesion and internal friction angle required by the model, while the unconfined compression tests provided the elastic modulus for describing deformation. In addition, the strength reduction method used for slope stability analysis is directly compatible with the Mohr-Coulomb shear strength parameters. The model is therefore appropriate for evaluating the global deformation, shear-strain localization, and factor of safety of the slope under different prescribed temperature-moisture conditions.

The numerical analysis was conducted as a prescribed-state seepage-deformation-stability assessment rather than as a fully coupled thermos-hydro-mechanical simulation. Temperature effects were introduced through the elastic modulus, cohesion, and internal friction angle measured after specimens had been conditioned at 23 ± 2°C, −5°C, and −15°C. Transient heat transfer, water–ice phase transformation, freezing-front migration, and the evolution of unfrozen water were not explicitly simulated. Accordingly, the numerical results represent comparisons among prescribed temperature and moisture states rather than the transient freezing process of the field slope.

The model was discretized using an unstructured mesh dominated by tetrahedral solid elements. The final finite element model contained 93,014 solid elements and 673,561 nodes. To improve computational accuracy in key regions, local mesh refinement was applied to the potential slip zone, the deformation-sensitive area near the slope toe, and the regions reserved for drainage pipe installation. The locally refined mesh regions are marked by black rectangles in the model figure. This meshing strategy balanced computational accuracy and efficiency and enabled the model to capture rainfall infiltration, pore-pressure variation, local deformation concentration, and the development of potential instability zones.

The same mesh was used for all temperature–moisture and rainfall cases to ensure consistency in the comparative analysis. Local refinement was applied to the potential slip zone, the slope-toe deformation-sensitive region, and the drainage-pipe installation zones. Model geometry, boundary continuity, and numerical convergence were checked before the multi-case calculations. The limit state was identified from numerical non-convergence, sudden displacement growth, or the formation of a continuous plastic zone.

The physical, hydraulic, and mechanical parameters used in the model were determined from field engineering geological investigations, laboratory tests, and engineering data from the study area. The upper and middle aquifers and the intervening aquitards were retained as separate spatial zones to reproduce the stratigraphic geometry, groundwater occurrence, and hydraulic-head distribution of the slope. The temperature-moisture-dependent elastic modulus, cohesion, and internal friction angle obtained from laboratory tests were assigned to the internal-dump rock-soil material domains represented by the sampled material. Other physical and hydraulic parameters were assigned according to the field investigation and hydrogeological data.

For each of the 12 temperature-moisture cases, the corresponding mean elastic modulus and the fitted cohesion and internal friction angle were used as equivalent continuum parameters. No additional empirical scale correction was applied. The laboratory parameters were not intended to reproduce every particle-scale heterogeneity within the full slope; instead, they were used to represent the average mechanical response of the internal-dump material under each prescribed environmental state. Because the same slope geometry, mesh, boundary conditions, and parameter-assignment procedure were used in all cases, the model primarily supports comparison of relative response trends among different temperature–moisture conditions. The absolute displacement and factor-of-safety values should therefore be interpreted as continuum-model estimates.

A permeability coefficient of 0.010368 m/day was used for all prescribed temperature states. This constant-permeability assumption was adopted to maintain a consistent hydraulic basis and isolate the influence of the temperature-dependent mechanical parameters on the comparative slope response. The model did not simulate water–ice phase transformation or temperature-dependent permeability reduction. In an actual frozen slope, pore-water freezing may reduce effective permeability and restrict rainfall infiltration. Therefore, using the same permeability coefficient at subzero temperatures may produce a relatively conservative estimate of rainfall penetration and rainfall-induced hydraulic disturbance. The subzero-temperature results should consequently be interpreted as comparative results under a prescribed permeability condition rather than as a complete description of frozen seepage behavior.

The model boundary conditions were defined according to the slope geometry and the requirements of finite element stability analysis. The bottom boundary of the model was constrained in both vertical and horizontal directions, whereas the lateral boundaries were constrained in the normal direction to reduce boundary interference with slope deformation. The slope surface was defined as a rainfall infiltration boundary, and a surface flux was applied under rainfall conditions. Hydraulic head boundaries were assigned at the interfaces between aquifers and aquitards according to groundwater occurrence conditions. To define the hydraulic boundary condition for the coupled seepage–stability analysis, regional precipitation characteristics and historical rainfall-intensity records were considered. Hydrological data published in 2023 indicate that the long-term mean annual precipitation in the area containing the Chaoyang open-pit coal mine is approximately 494.5 mm. On this basis, and considering the potential threat of sustained extreme rainfall to the internal dump slope, a prescribed surface infiltration flux of 0.05 m^3^/(m^2^·day), equivalent to 0.05 m/day or 50 mm/day, was applied for 72 h. The corresponding cumulative rainfall input was 150 mm.

This boundary condition represents a continuous heavy-rainfall scenario selected from the regional hydrological background and historical rainfall-intensity records. It was used to evaluate the operation of the drainage system and the seepage-deformation-stability response of the slope under an unfavorable rainfall condition. The same rainfall flux and duration were applied to all temperature–moisture cases to provide a consistent basis for comparison.

The adopted event should be regarded as a representative engineering scenario rather than a site-specific design storm or a critical rainfall threshold. The present study did not systematically compare multiple combinations of rainfall intensity and duration; therefore, the numerical results primarily indicate the relative responses of different temperature–moisture states under the prescribed rainfall boundary.

To describe the variation in pore water pressure within the slope during rainfall infiltration, a saturated-unsaturated seepage analysis was adopted. The seepage field was described using the total hydraulic head, which can be expressed as:


$$\begin{array}{c} h=z+\frac{u_{w}}{\gamma_{w}} \end{array}$$ (2)

where *h* is the total hydraulic head, *z* is the elevation head, *u*_w_ is the pore water pressure, and *γ*_w_ is the unit weight of water. Therefore, the pore water pressure can be calculated from the difference between the total hydraulic head and the elevation head:


$$\begin{array}{c} u_{w}=\gamma_{w}\left(h-z\right) \end{array}$$ (3)

In the unsaturated zone, *u*_w_ may be negative, representing matric suction, whereas in the saturated zone, *u*_w_ is generally positive. Rainfall infiltration changes the hydraulic head distribution within the slope and further induces redistribution of the pore water pressure field. The change in pore water pressure affects the shear strength of the soil through effective stress, which can be expressed as:


$$\begin{array}{c} \sigma^{\prime }\;=\sigma -u_{w} \end{array}$$ (4)

where *σ*′ is the effective normal stress and *σ* is the total normal stress. According to the Mohr–Coulomb strength criterion, an increase in pore water pressure reduces the effective stress and consequently weakens the shear strength of the soil:


$$\begin{array}{c} \tau_{f}\;=c\prime +(\sigma \;-u_{w})tan\varphi \prime \end{array}$$ (5)

where *τ*_f_ is the shear strength, *c*′ is the effective cohesion, and *φ*′ is the effective internal friction angle. Thus, the increase in pore water pressure induced by rainfall infiltration is one of the key hydraulic factors responsible for reducing slope stability.

The numerical analysis procedure included initial seepage analysis, initial stress equilibrium analysis, rainfall seepage analysis, post-rainfall stress–deformation analysis, and strength reduction stability analysis. First, the initial groundwater seepage field was established, followed by an initial stress equilibrium calculation. Then, the rainfall infiltration boundary with a duration of 72 h was applied to conduct transient seepage analysis under rainfall conditions. After rainfall, the pore water pressure field obtained from the seepage analysis was transferred to the stress–deformation analysis module to calculate slope displacement and strain responses. Finally, the strength reduction method was used to determine the safety factors before and after rainfall.

The strength reduction method determines the factor of safety(FOS) by progressively reducing the shear strength parameters of the material until the slope reaches a limit equilibrium state. According to the Mohr–Coulomb strength criterion, the reduced cohesion and internal friction angle can be expressed as:


$$\begin{array}{c} \left\{ \begin{array}{c} @l@\;c_{r}=\frac{c}{F_{s}}\\ \varphi_{r}=arctan\left(\frac{tan\varphi }{F_{s}}\right) \end{array}\right. \end{array}$$ (6)

where *c*_r_ and *φ*_r_ are the reduced cohesion and internal friction angle, respectively; *c* and *φ* are the original cohesion and internal friction angle obtained from laboratory tests, respectively; and *F*_s_ is the strength reduction factor. When numerical non-convergence, a sudden increase in displacement at characteristic points, or the formation of a continuous plastic zone occurs, the slope is considered to have reached the limit state. The corresponding reduction factor is then taken as the FOS of the slope.

To systematically analyze the coupled effects of temperature, moisture content, and rainfall on slope stability, 12 temperature–moisture numerical simulation cases were established. The moisture contents included 14%, natural moisture content of 17.6%, 23%, and 26%, and the temperatures included RT, −5°C, and −15°C. For each case, the material parameters were assigned according to the corresponding laboratory test results, and the safety factors before and after rainfall were calculated. This multi-case simulation framework enables the independent and coupled effects of temperature and moisture content on slope stability to be clarified and provides a basis for evaluating rainfall-induced stability of internal dump slopes in cold regions.

### 3.6. HDPE perforated drainage pipe simulation and field monitoring

**Fig 7:**
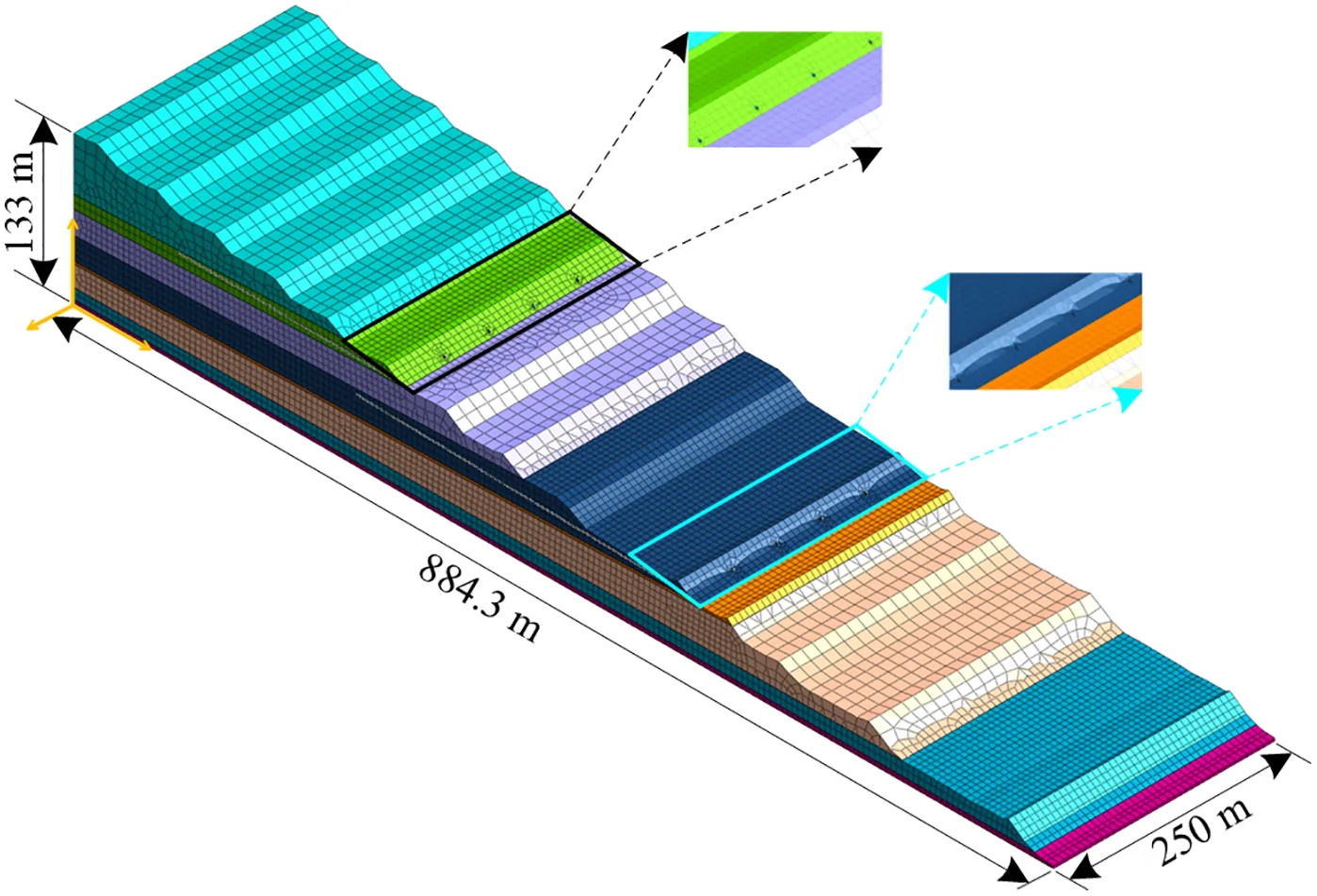
HDPE perforated drainage pipe model for the internal dump slope.

To evaluate the influence of drainage measures on the seepage field and stability of the internal dump slope, HDPE perforated drainage pipes were incorporated into the original GTS NX slope model, and drainage simulations were conducted under rainfall conditions. The drainage pipes were mainly arranged in the upper and middle aquifers to simulate the influence of engineering drainage measures on groundwater discharge within the slope [21]. As shown in Fig 7, the drainage pipes were primarily arranged were primarily arranged in the upper and middle aquifers because these two units are the principal groundwater-bearing layers controlling pore water pressure within the slope. The upper aquifer has an average thickness of approximately 11.75 m and a permeability coefficient of 10.447 m/day, whereas the middle aquifer has a greater average thickness of approximately 21.9 m and a permeability coefficient of 1.344 m/day. The upper aquifer provides a relatively active groundwater migration pathway, while the thicker middle aquifer represents an important groundwater-storage and pressure-control unit.

In contrast, the lower aquifer is relatively thin, discontinuous, and locally absent, and its influence is partly separated by the lower aquitard. The upper and middle aquifers were therefore selected as the primary drainage targets because reducing their hydraulic head and pore water pressure was expected to produce the most direct improvement in the internal hydraulic condition of the slope. The pipe lengths of 200 m and 300 m were selected to intersect the corresponding upper and middle water-bearing zones and provide continuous discharge paths toward the slope drainage system.

**Fig 8:**
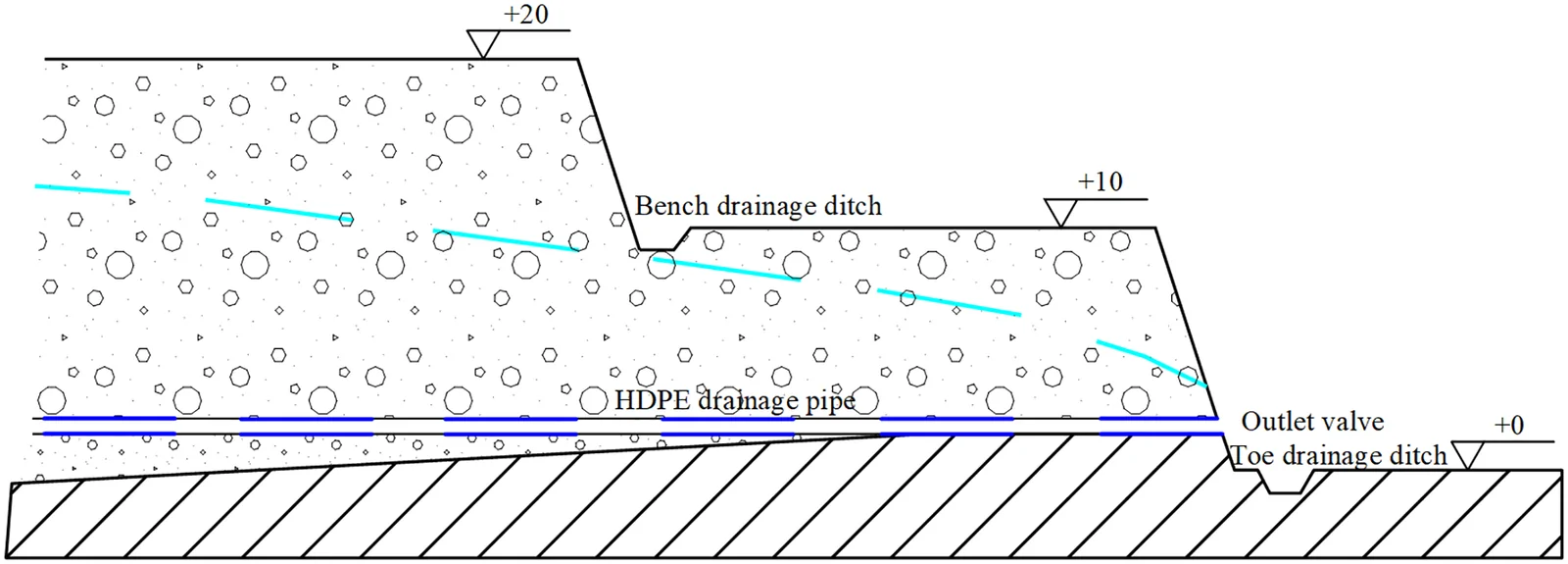
Cross-sectional view of the drainage hole installation.

In the numerical model, the drainage pipe length was set to 200 m in the upper aquifer and 300 m in the middle aquifer. The pipe diameter was 0.13 m, and the pipe spacing was 40 m. The material parameters of the HDPE perforated drainage pipe included an elastic modulus of 100000 kN/m^2^, a Poisson’s ratio of 0.42, a unit weight of 19.1 kN/m^3^, and a permeability coefficient of 0.864 m/day. The same rainfall boundary condition as that used in the original slope model was adopted, with a rainfall infiltration flux of 0.05 m/day and a rainfall duration of 3 days, i.e., 72 h. The cross-sectional view is shown in Fig 8.

The laboratory tests were designed to determine the bulk mechanical parameters of the internal-dump rock-soil mixture. Drainage holes were not introduced into the 61.8 mm laboratory specimens because a geometrically scaled hole would occupy a substantial proportion of the specimen cross-section and would introduce strong size and boundary effects. Such a specimen would characterize a local perforated structure rather than the representative bulk material. The drainage components were therefore considered at the slope-model scale instead of being incorporated into the material-parameter tests.

In the numerical model, the HDPE perforated drainage pipes were represented as discrete drainage components with their own elastic and hydraulic parameters. Their installation did not alter the Mohr-Coulomb constitutive parameters of the surrounding bulk material. The drainage effect entered the model through the geometry and properties of the drainage components and the resulting redistribution of the seepage and stress fields.

The stiffness difference between the HDPE component and the surrounding rock-soil material may lead to localized stress or plastic-strain concentration near the drainage-pipe region. Such local concentrations were therefore distinguished from a continuous plastic band controlling global slope failure. The present model represents the stiffness contrast at the continuum level but does not explicitly simulate installation disturbance, detailed perforation geometry, pipe-soil interface slip, or progressive interface separation. The calculated pipe-adjacent plastic zones should consequently be interpreted as local numerical responses rather than direct evidence of an overall slip surface.

To verify the engineering applicability of the drainage measure in the numerical simulation, field monitoring was conducted in combination with the drainage project on the north slope of the internal dump in the Chaoyang open-pit coal mine. DN400 HDPE perforated drainage pipes were installed in the upper and middle aquifers, and groundwater observation holes were arranged to monitor the average daily drainage volume and groundwater-level variations in the upper and middle aquifers during drainage system operation.

The field monitoring program lasted for one month and focused on the hydraulic performance of the drainage system. The monitored variables included the average daily drainage volume and groundwater-level changes in the upper and middle aquifers. Dedicated slope-displacement monitoring, such as inclinometers, GNSS measurements, or surface crack gauges, was not included in the available monitoring dataset. Therefore, the field observations were used to validate the groundwater-drainage and drawdown effects rather than to directly validate the calculated displacement or plastic-strain fields.

## 4. Results

### 4.1. Unconfined compression behavior under different temperature–moisture conditions

**Fig 9:**
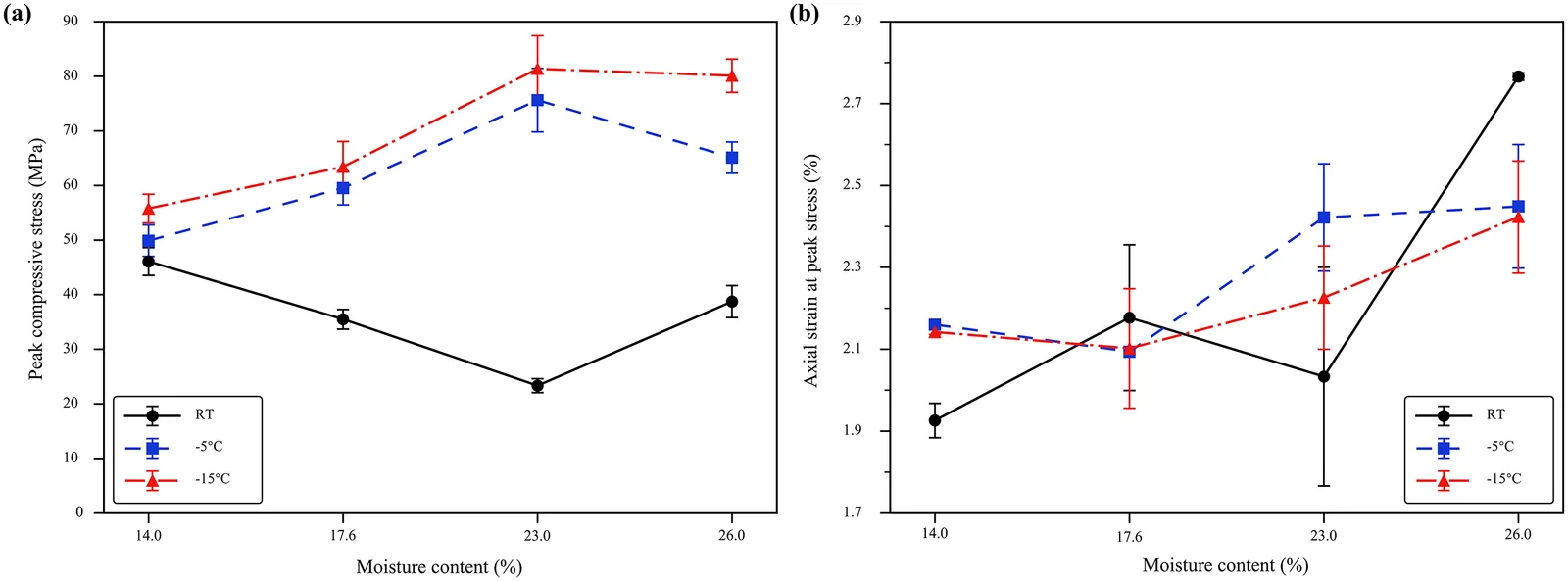
Peak compressive response of internal-dump material specimens under different temperature–moisture conditions. (a) Mean peak compressive stress, (b) mean axial strain corresponding to peak stress.

The unconfined compression tests were conducted to investigate the strength and deformation responses of the internal-dump rock–soil mixture under different temperature–moisture conditions. Fig 9 summarizes the mean peak compressive stress and the corresponding axial strain for each condition. The error bars represent one standard deviation calculated from three independent specimens. Table 1 summarizes the elastic modulus results under the different temperature–moisture conditions.

**Table 1:** Elastic modulus of internal-dump material specimens under different temperature-moisture conditions. Values are expressed as mean ± standard deviation (*n* = 3).

Moisture content	RT (MPa)	−5°C (MPa)	−15°C (MPa)
14.0%	1802.4 ± 10.5	2201.8 ± 7.1	2501.8 ± 7.9
17.6%	1502.4 ± 9.4	2502.6 ± 8.1	2999.1 ± 13.3
23.0%	1200.7 ± 6.6	2802.3 ± 6.7	3502.1 ± 7.1
26.0%	1003.6 ± 6.9	2503.8 ± 6.9	3203.3 ± 7.4

At RT, the elastic modulus decreased continuously with increasing moisture content. The mean elastic modulus decreased from 1802.4 MPa at 14% moisture content to 1502.4 MPa at the natural moisture content, 1200.7 MPa at 23% moisture content, and 1003.6 MPa at 26% moisture content. Compared with the 14% moisture-content condition, the elastic modulus decreased by approximately 44.3% at 26% moisture content. This result indicates that, under unfrozen conditions, increasing moisture content significantly weakened the stiffness of the internal-dump material.

Under subzero temperatures, the elastic modulus increased markedly compared with that at RT. At −5°C, the mean elastic modulus increased from 2201.8 MPa at 14% moisture content to 2502.6 MPa at the natural moisture content and reached 2802.3 MPa at 23% moisture content, before decreasing to 2503.8 MPa at 26% moisture content. A similar trend was observed at −15°C, where the elastic modulus increased from 2501.8 MPa at 14% moisture content to 2999.1 MPa at the natural moisture content and reached the maximum value of 3502.1 MPa at 23% moisture content, followed by a decrease to 3203.3 MPa at 26% moisture content.

A comparison among the three temperature levels shows that decreasing temperature generally increased the elastic modulus of the specimens. This strengthening effect was more evident at higher moisture contents. For example, at 23% moisture content, the elastic modulus increased from 1200.7 MPa at RT to 2802.3 MPa at −5°C and 3502.1 MPa at −15°C. At 26% moisture content, the elastic modulus increased from 1003.6 MPa at RT to 2503.8 MPa at −5°C and 3203.3 MPa at −15°C. These results demonstrate that the mechanical response of the internal-dump material is strongly controlled by the coupling between temperature and moisture content.

Overall, the unconfined compression results reveal two distinct temperature-dependent responses. At RT, increasing moisture content continuously reduced specimen stiffness. Under −5°C and −15°C conditions, the elastic modulus increased from low to intermediate moisture contents and reached its highest measured value at 23.0% moisture content, followed by a moderate decrease at 26.0%. These results demonstrate that the mechanical effect of moisture depends strongly on its phase state and temperature condition.

### 4.2. Shear strength characteristics under different temperature–moisture conditions

**Fig 10:**
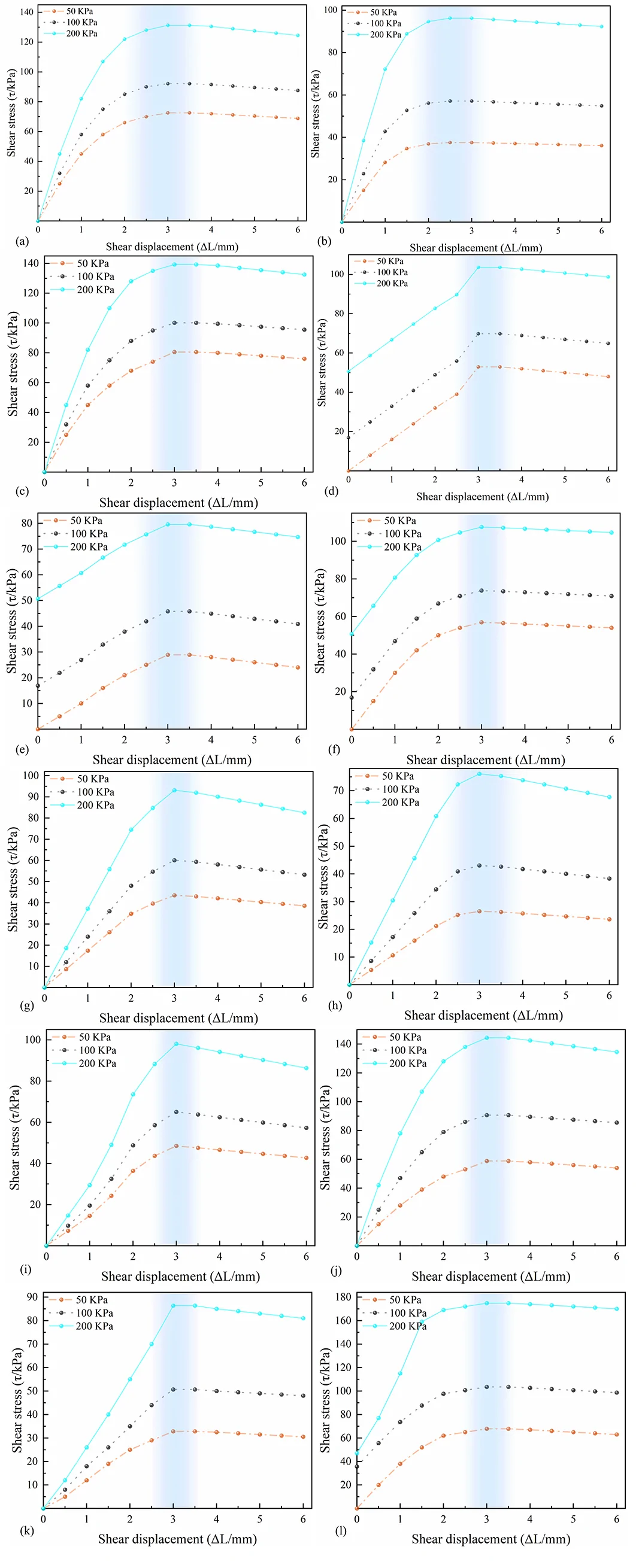
Shear stress–shear displacement curves of internal-dump material specimens under different temperature and moisture-content conditions. 14% moisture content, −5°C, 14% moisture content, RT, 14% moisture content, −15°C, 23% moisture content, −5°C, 23% moisture content, RT, 23% moisture content, −15°C, 26% moisture content, −5°C, 26% moisture content, RT, 26% moisture content, −15°C, Natural moisture content, −5°C, Natural moisture content, RT, Natural moisture content, −15°C.

**Fig 11:**
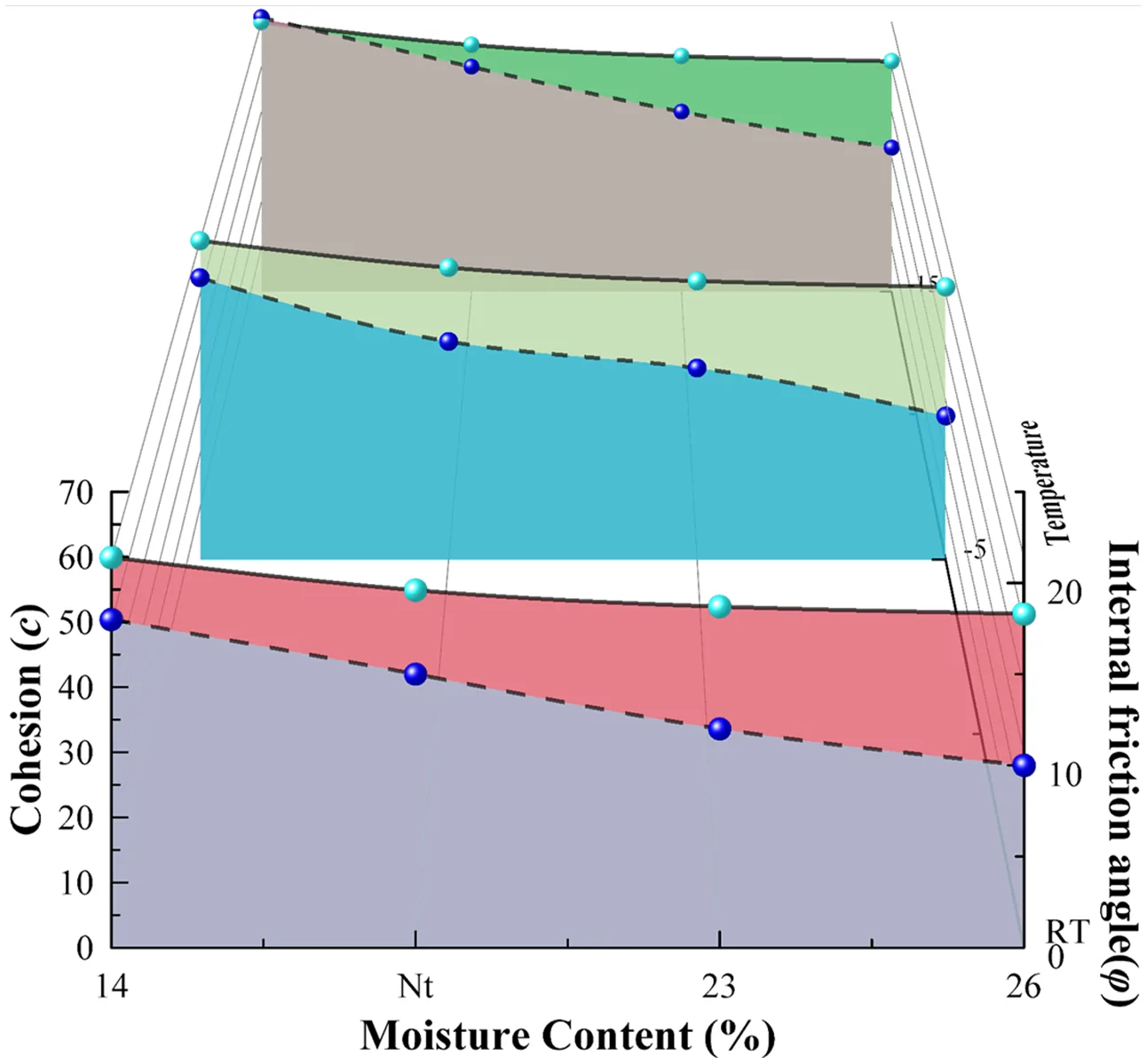
Variation characteristics of cohesion and internal friction angle of internal-dump rock–soil mixture specimens under different temperature and moisture-content conditions.

Direct shear tests were conducted to determine the shear strength response of the internal-dump rock–soil mixture under different temperature–moisture conditions. Fig 10 presents the shear stress–displacement curves obtained under normal stresses of 50, 100, and 200 kPa. Table 2 summarizes the independently fitted cohesion and internal friction angle for each temperature–moisture condition, while Fig 11 illustrates the variation in these shear strength parameters. Overall, both moisture content and temperature influenced the shear strength of the specimens. Cohesion was more sensitive to temperature variation, whereas the internal friction angle mainly decreased with increasing moisture content.

**Table 2:** Cohesion and internal friction angle of internal-dump material specimens under different temperature and moisture-content conditions.

Moisture Content	Test Temperature					
	RT		−5°C		−15°C	
	Cohesion(*c*)	Internal friction angle(*φ*)	Cohesion(*c*)	Internal friction angle(*φ*)	Cohesion(*c*)	Internal friction angle(*φ*)
	/kPa	/°	/kPa	/°	/kPa	/°
14%	18	23.43	53	21.06	61	21.97
Natural	15	20.05	41	19.58	50	19.54
23%	12	18.65	36	19.23	40	19.12
26%	10	18.11	27	17.87	32	18.62

At RT, cohesion decreased continuously with increasing moisture content. The cohesion decreased from 18 kPa at 14% moisture content to 15 kPa at the natural moisture content, 12 kPa at 23% moisture content, and 10 kPa at 26% moisture content. Compared with the 14% moisture-content condition, the cohesion decreased by approximately 44.4% at 26% moisture content. This result indicates that increasing moisture content weakened the interparticle bonding and shear resistance of the rock–soil mixture specimens under unfrozen conditions.

Under subzero-temperature conditions, cohesion increased significantly compared with that at RT. At −5°C, the cohesion values of the specimens with 14%, natural, 23%, and 26% moisture contents were 53, 41, 36, and 27 kPa, respectively. At −15°C, the corresponding cohesion values further changed to 61, 50, 40, and 32 kPa. Compared with the room-temperature condition, higher cohesion was observed under subzero temperatures at the same moisture content. For example, at 14% moisture content, cohesion increased from 18 kPa at RT to 53 kPa at −5°C and 61 kPa at −15°C. At 26% moisture content, cohesion increased from 10 kPa at RT to 27 kPa at −5°C and 32 kPa at −15°C.

Compared with cohesion, the internal friction angle exhibited a smaller and non-monotonic response to temperature. At a moisture content of 14.0%, the internal friction angle was 23.43° at RT, 21.06° at −5°C, and 21.97° at −15°C. At the natural moisture content of 17.6%, the corresponding values were 20.05°, 19.58°, and 19.54°, respectively. At moisture contents of 23.0% and 26.0%, the internal friction angles ranged from 18.65° to 19.23° and from 17.87° to 18.62°, respectively.

As shown in Fig 11, no monotonic relationship was observed between temperature and internal friction angle. was observed between temperature and internal friction angle. Depending on moisture content, cooling either slightly increased or decreased the fitted friction angle. In contrast, increasing moisture content produced a consistent overall reduction in the internal friction angle at all three temperatures. From 14.0% to 26.0% moisture content, the internal friction angle decreased from 23.43° to 18.11° at RT, from 21.06° to 17.87° at −5°C, and from 21.97° to 18.62° at −15°C.

These results indicate that the internal friction angle was controlled primarily by moisture-dependent changes in particle contact, interlocking, and frictional sliding, whereas the temperature effect was comparatively limited and non-monotonic. Temperature reduction had a much stronger influence on cohesion than on the internal friction angle.

### 4.3. Seepage–deformation response of the slope before and after rainfall under 14% moisture content at RT

**Fig 12:**
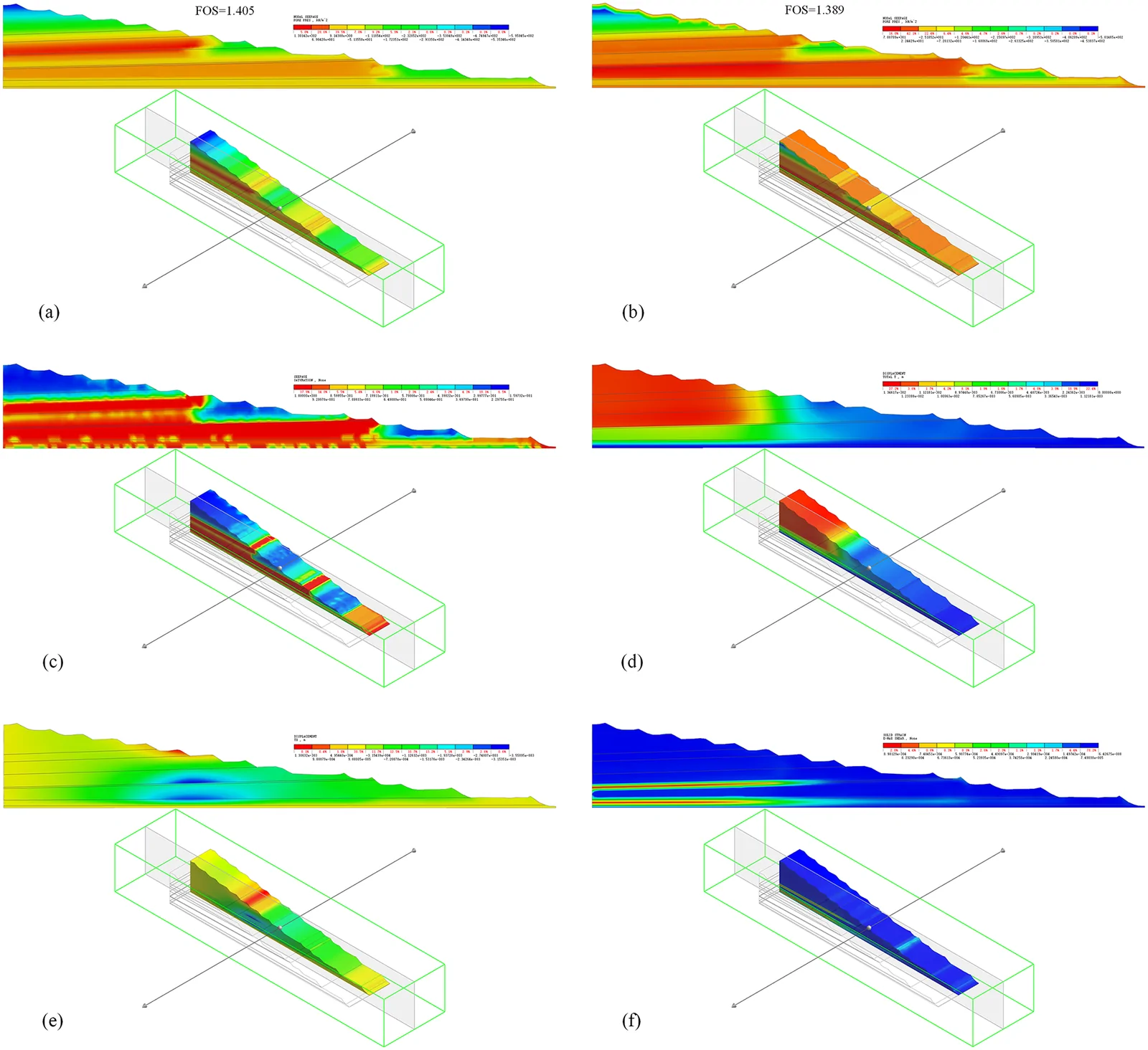
Seepage–deformation response characteristics of the internal dump slope before and after rainfall under 14% moisture content at RT. (a) Pore water pressure before rainfall, (b) Pore water pressure after rainfall, (c) Saturation after rainfall, (d) Total displacement after rainfall, (e) Horizontal displacement after rainfall, (f) Maximum shear strain after rainfall.

Based on the laboratory-derived mechanical parameters, the condition with 14% moisture content at RT was selected as a representative case to further analyze the seepage, displacement, and strain responses of the internal dump slope under rainfall. Fig 12 shows the simulated pore water pressure, saturation, displacement, and maximum shear strain distributions before and after rainfall under this condition.

Before rainfall, the pore water pressure in the slope showed a clear spatial zonation, ranging from approximately −595.85 to 130.14 kPa. Negative pore water pressure was mainly distributed in the upper part and near-surface region of the slope, indicating that a certain matric suction still existed in these areas. In contrast, relatively higher pore water pressure was observed in the middle and lower parts of the slope, reflecting the nonuniform distribution of water within the slope under the initial hydraulic boundary conditions. Overall, no extensive continuous high-pore-pressure zone was formed before rainfall, and the internal hydraulic state of the slope remained relatively stable.

After rainfall, the pore water pressure ranged from −501.47 to 70.07 kPa. Compared with the pre-rainfall condition, the absolute value of negative pore water pressure decreased, indicating that rainfall infiltration weakened the matric suction in the shallow and upper parts of the slope and induced a redistribution of the pore pressure field. Spatially, the change in pore water pressure was mainly concentrated in the shallow slope, local bench platforms, and the middle-to-lower water-bearing zones, suggesting that the influence of rainfall infiltration on the hydraulic state of the slope was spatially nonuniform.

The saturation contour after rainfall further reflects the redistribution of water within the slope. The simulated saturation ranged from approximately 0.16 to 1.00 after rainfall. High-saturation zones were mainly distributed in the middle and lower parts of the slope, local bench platforms, and the bottom region. The slope did not become fully saturated as a whole, indicating that rainfall infiltration increased saturation only in localized areas under the initial moisture content of 14%, without forming an extensive continuous saturated zone.

Under the rainfall-induced stress field, the maximum total displacement of the slope was approximately 3.46 × 10^−2^ m. The high-displacement zone was mainly distributed in the left high-bench or high-fill region and gradually decreased toward the lower benches and slope toe. This indicates that the slope experienced a certain deformation response after rainfall, but the deformation did not show a continuous overall sliding pattern. Instead, it was mainly characterized by local settlement, compression deformation, and internal stress adjustment. Further analysis of the X-direction horizontal displacement showed that it ranged from approximately −3.56 × 10^−3^ to 1.31 × 10^−3^ m, remaining at the millimeter scale and being much smaller than the total displacement. This suggests that no significant overall horizontal sliding occurred under this condition.

The maximum shear strain distribution showed localized shear strain concentration within the slope after rainfall, with a maximum value of approximately 8.98 × 10^−4^. The high-shear-strain zones were mainly distributed along local strata or near the bottom region, without forming a continuous shear band extending from the rear part of the slope to the slope toe. This result indicates that rainfall induced local shear deformation concentration but did not lead to the through-going development of a potential slip surface.

The strength reduction results further show that the FOS under 14% moisture content at RT decreased from 1.4047 before rainfall to 1.3891 after rainfall. Based on the rounded values listed in the table, the FOS decreased from 1.405 to 1.390, corresponding to a reduction of approximately 1.07%; using the original model outputs, the reduction was approximately 1.1%. This indicates that rainfall infiltration had an adverse but relatively limited effect on slope stability. Considering the pore water pressure, saturation, displacement, maximum shear strain, and FOS results together, the slope under 14% moisture content at RT remained globally stable after rainfall, without obvious overall sliding deformation or a through-going shear failure surface.

### 4.4. Variation in slope safety factor under coupled temperature–moisture–rainfall conditions

To reveal the coupled effects of temperature, moisture content, and rainfall on the global stability of the internal dump slope, the factors of safety before and after rainfall were calculated for the 12 numerical cases using the corresponding laboratory-derived mechanical parameters. Table 3 summarizes the factors of safety and rainfall-induced reduction rates. Although all calculated factors of safety were greater than 1.0, their absolute values and sensitivities to rainfall differed substantially among the temperature–moisture conditions.

**Table 3:** Safety factors and reduction rates of the internal dump slope before and after rainfall under different temperature and moisture-content conditions.

Temperature	Moisture Content	Before Rainfall	After Rainfall	Reduction rate
RT	14%	1.405	1.390	1.07%
	Natural	1.221	1.184	3.03%
	23%	1.203	1.174	2.41%
	26%	1.176	1.166	0.85%
−5°C	14%	1.476	1.469	0.47%
	Natural	1.493	1.485	0.54%
	23%	1.515	1.513	0.13%
	26%	1.535	1.533	0.13%
−15°C	14%	1.484	1.478	0.40%
	Natural	1.505	1.499	0.40%
	23%	1.521	1.518	0.20%
	26%	1.540	1.537	0.19%

At RT, the safety factor showed an overall decreasing trend with increasing moisture content. Before rainfall, the safety factor decreased from 1.405 at 14% moisture content to 1.221 at the natural moisture content, 1.203 at 23% moisture content, and 1.176 at 26% moisture content. After rainfall, the corresponding safety factor decreased from 1.390 to 1.184, 1.174, and 1.166, respectively. These results indicate that, under unfrozen conditions, increasing moisture content significantly reduced the stability reserve of the slope, and the slope under high moisture-content conditions was closer to a critical stable state.

Different from the room-temperature condition, the safety factor was generally higher under subzero temperatures and increased with increasing moisture content. At −5°C, the safety factor before rainfall increased from 1.476 at 14% moisture content to 1.535 at 26% moisture content, while the safety factor after rainfall increased from 1.469 to 1.533. At −15°C, the safety factor further increased slightly, from 1.484 to 1.540 before rainfall and from 1.478 to 1.537 after rainfall. These results indicate that subzero temperature changed the role of moisture content in slope stability, causing the slope with higher moisture content to exhibit a higher safety factor.

The rainfall-induced reduction in factor of safety did not increase monotonically with the initial moisture content. At RT, the factor of safety decreased from 1.221 to 1.184 under the natural moisture condition, corresponding to a reduction of 3.03%, whereas it decreased from 1.176 to 1.166 at 26% moisture content, corresponding to a reduction of 0.85%. These results describe two different aspects of slope response. The 26% moisture-content condition had the lowest initial and post-rainfall factors of safety and therefore represented the smallest overall stability reserve. In contrast, the natural moisture condition exhibited the largest incremental response to rainfall.

This difference can be attributed to the initial hydraulic state. At the natural moisture content, part of the matric-suction contribution remained before rainfall, while the material was sufficiently wet to permit marked water migration and local saturation development. Rainfall therefore caused a relatively large loss of suction and effective stress. At 26% moisture content, the material was already in a highly unfavorable hydraulic and mechanical state before rainfall, with lower stiffness and shear strength and less remaining suction contribution. Consequently, the additional reduction caused by the prescribed 72 h rainfall was smaller, although the absolute post-rainfall factor of safety remained the lowest. Thus, the largest rainfall-induced percentage reduction does not necessarily correspond to the lowest final stability level.

Within the strength-reduction framework used in this study, a factor of safety greater than 1.0 indicates that the numerical model had not reached the defined limit state under the prescribed loading and hydraulic conditions. However, this does not mean that all cases have the same engineering safety margin or that a value slightly greater than 1.0 automatically satisfies every project-specific design requirement.

The absolute factor of safety and the rainfall-induced reduction should therefore be interpreted together. The absolute value reflects the remaining global stability reserve, whereas the reduction before and after rainfall reflects the sensitivity of that reserve to hydraulic disturbance. For example, the 26% moisture-content case had the lowest post-rainfall factor of safety, while the natural moisture case experienced the largest rainfall-induced reduction. Both indicators are relevant for identifying unfavorable states and rainfall-sensitive conditions.

Overall, the simulation results under different temperature and moisture-content conditions show that RT represents the most unfavorable environmental condition for slope stability under rainfall, especially under the natural and 23% moisture-content conditions, where the reduction in safety factor was more pronounced. Under subzero temperatures, the safety factor increased overall, and the difference before and after rainfall was relatively small, indicating that the sensitivity of the slope to short-term rainfall disturbance decreased under frozen conditions. Therefore, temperature not only affects the strength parameters of the slope material but also changes the way in which moisture content and rainfall influence slope stability.

### 4.5. Drainage effect and field validation of HDPE perforated drainage pipes

**Fig 13:**
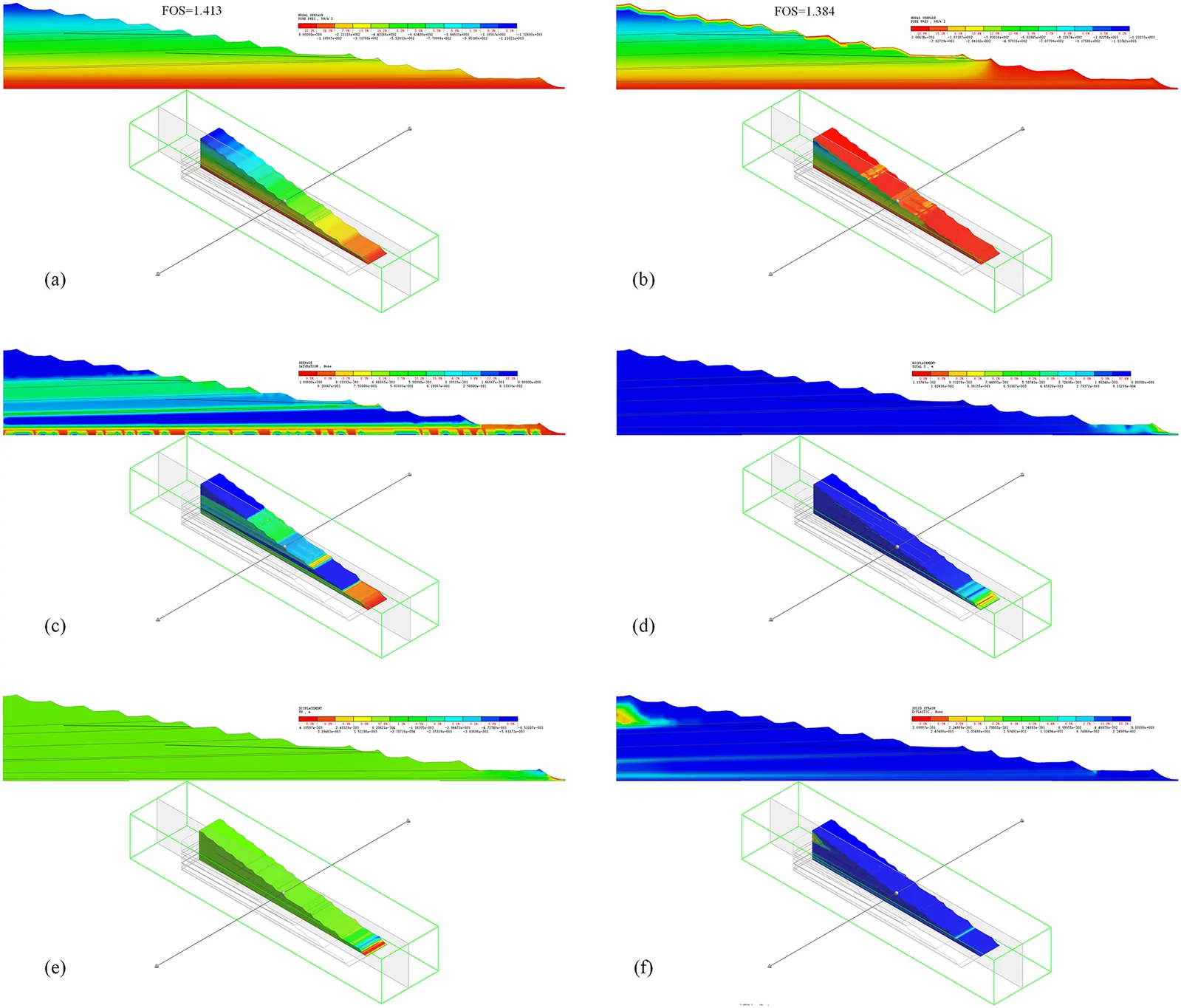
Seepage–deformation response characteristics of the internal dump slope with HDPE perforated drainage pipes under rainfall conditions. (a) Pore water pressure before rainfall, (b) Pore water pressure after rainfall, (c) Saturation after rainfall, (d) Total displacement after rainfall, (e) Horizontal displacement after rainfall, (f) Effective plastic strain after rainfall.

Fig 13 shows the seepage-deformation response of the slope with HDPE perforated drainage pipes under rainfall conditions. The pore water pressure distributions before and after rainfall indicate that the internal hydraulic field of the slope exhibited a clear drainage response after the installation of the drainage pipes. Before rainfall, pore water pressure was mainly controlled by the initial groundwater level and aquifer distribution, and the hydraulic gradient was relatively gentle. After rainfall, pore water pressure changed in the shallow slope and around local bench platforms, but the high-pore-pressure zone did not expand extensively within the slope. This indicates that the HDPE perforated drainage pipes provided preferential drainage pathways for groundwater, allowing local water pressure induced by rainfall infiltration to be released through the drainage pipes and thereby reducing pore-pressure accumulation within the slope.

The saturation distribution after rainfall further indicates that the drainage pipes regulated the redistribution of water within the slope. The simulation results show that the high-saturation zones after rainfall were mainly confined to local aquifers, areas around the drainage pipes, and low-lying parts of the slope, without forming an extensive continuous saturated zone. This result suggests that the HDPE perforated drainage pipes restricted the expansion of the saturated zone within the slope after rainfall infiltration, thereby reducing the possibility of strength degradation induced by increasing moisture content.

The displacement field results show that, after the installation of drainage pipes, the total displacement and horizontal displacement of the slope after rainfall were mainly concentrated near the local slope toe, lower benches, and pipe-adjacent areas, whereas the main body of the slope exhibited relatively small displacement. No continuous overall sliding deformation developed along the slope surface or within the slope. This result indicates that the overall deformation of the slope was restrained to a certain extent after the installation of drainage pipes. The horizontal displacement results also show that no large-scale horizontal movement toward the free face occurred, confirming that the slope remained globally stable under rainfall conditions.

The effective plastic strain distribution further reflects the influence of drainage pipes on potential failure zones. The simulation results show that the effective plastic strain was low in most parts of the slope after rainfall, with strain concentration occurring only near the local slope toe, drainage pipes, or geometrical transition zones. The high-plastic-strain zones did not develop into a continuous plastic band extending from the rear part of the slope to the slope toe. This indicates that, although local plastic strain concentration occurred within the slope after the installation of HDPE perforated drainage pipes, it did not develop into a through-going slip surface controlling global slope stability.

The strength reduction results showed that, under the room-temperature natural moisture-content condition, the safety factor of the slope without drainage pipes was 1.221 before rainfall and decreased to 1.184 after rainfall. After installing HDPE perforated drainage pipes, the safety factor increased to 1.413 before rainfall and remained at 1.384 after rainfall. Compared with the case without drainage pipes, the safety factors before and after rainfall increased by approximately 15.72% and 16.89%, respectively. In addition, the rainfall-induced reduction in safety factor decreased from 3.03% to approximately 2.05%. These results indicate that HDPE perforated drainage pipes significantly improved the stability of the slope under the room-temperature natural moisture-content condition and reduced the sensitivity of the slope to rainfall disturbance.

**Fig 14:**
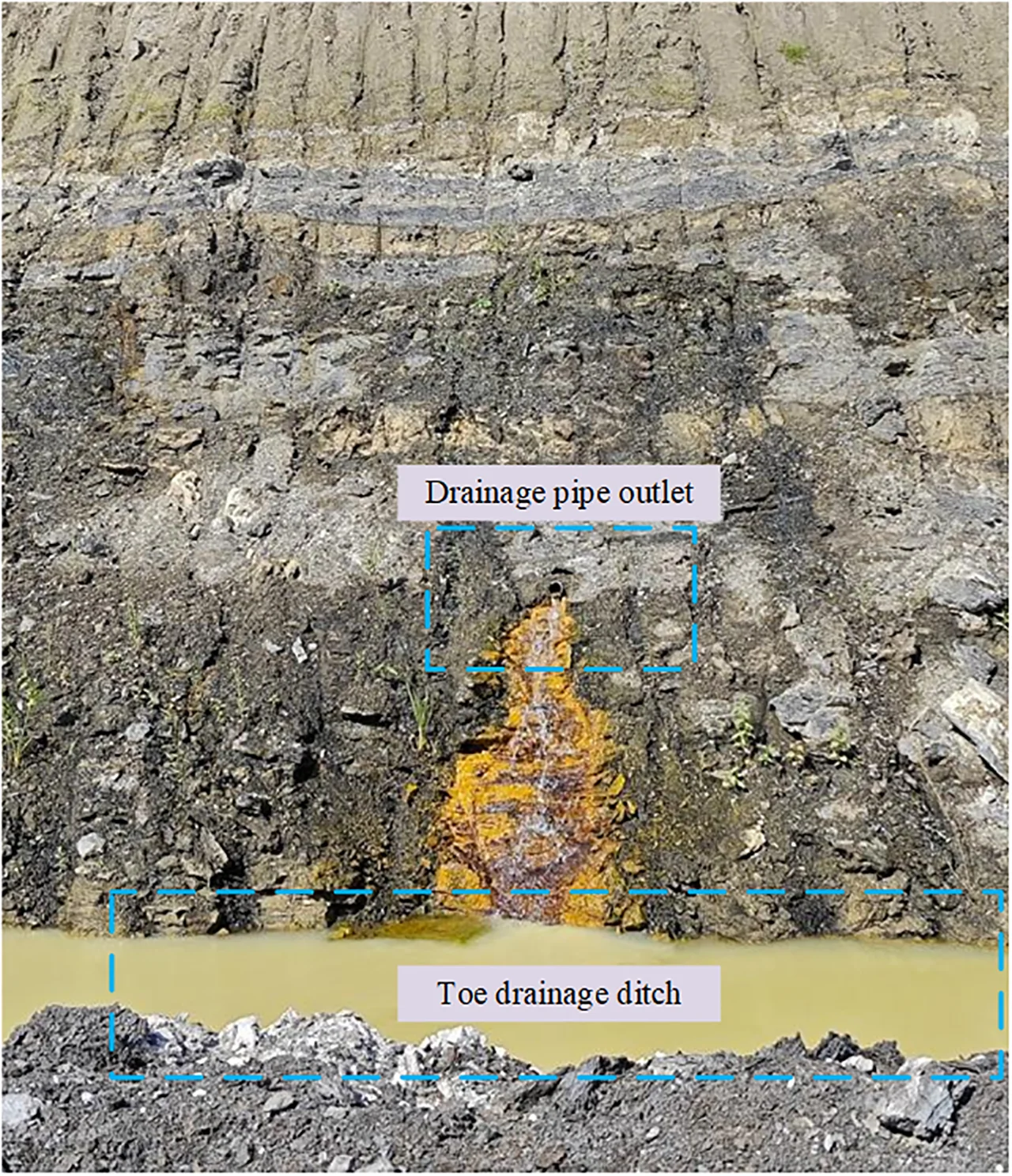
Field outlet of the HDPE perforated drainage pipe and the toe drainage ditch. The photograph was taken and annotated by the authors.

**Fig 15:**
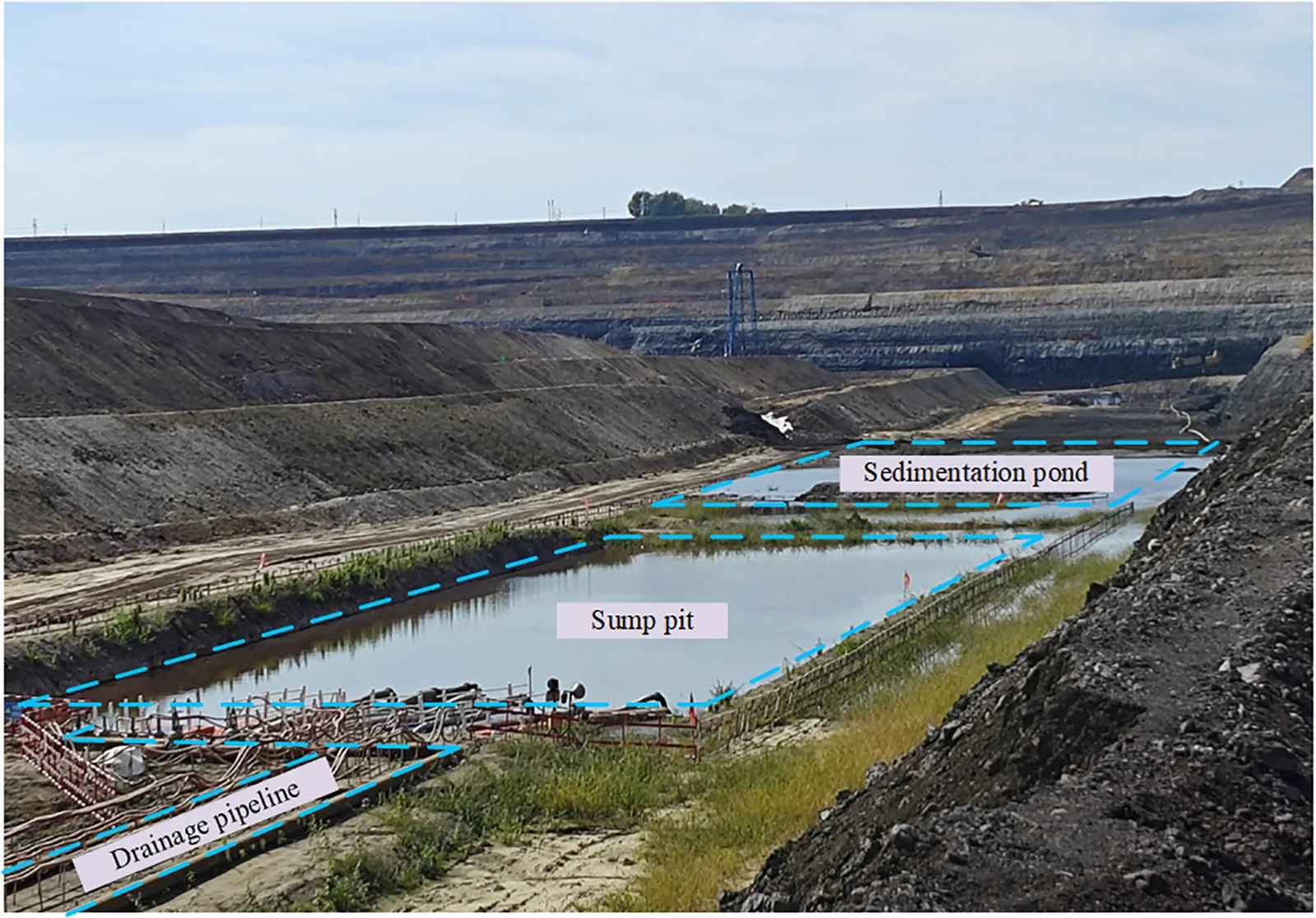
Field sump, sedimentation pond, and drainage pipeline. The photograph was taken and annotated by the authors.

To verify the engineering applicability of the drainage measure in the numerical simulation, field drainage validation was further conducted at the north slope of the internal dump in the Chaoyang open-pit coal mine. DN400 HDPE perforated drainage pipes were installed in the upper and middle aquifers of the slope, as shown in Fig 14, and observation holes were arranged to monitor changes in groundwater level. The field drainage system collected seepage water from the slope through the drainage pipes, conveyed it to the sump at the pit bottom through drainage ditches along the slope toe, as shown in Fig 15, and then transported it to a modular water-treatment system using submersible pumps and pressure pipelines. After treatment, the water was discharged into the drainage-water regulation pond.

**Fig 16:**
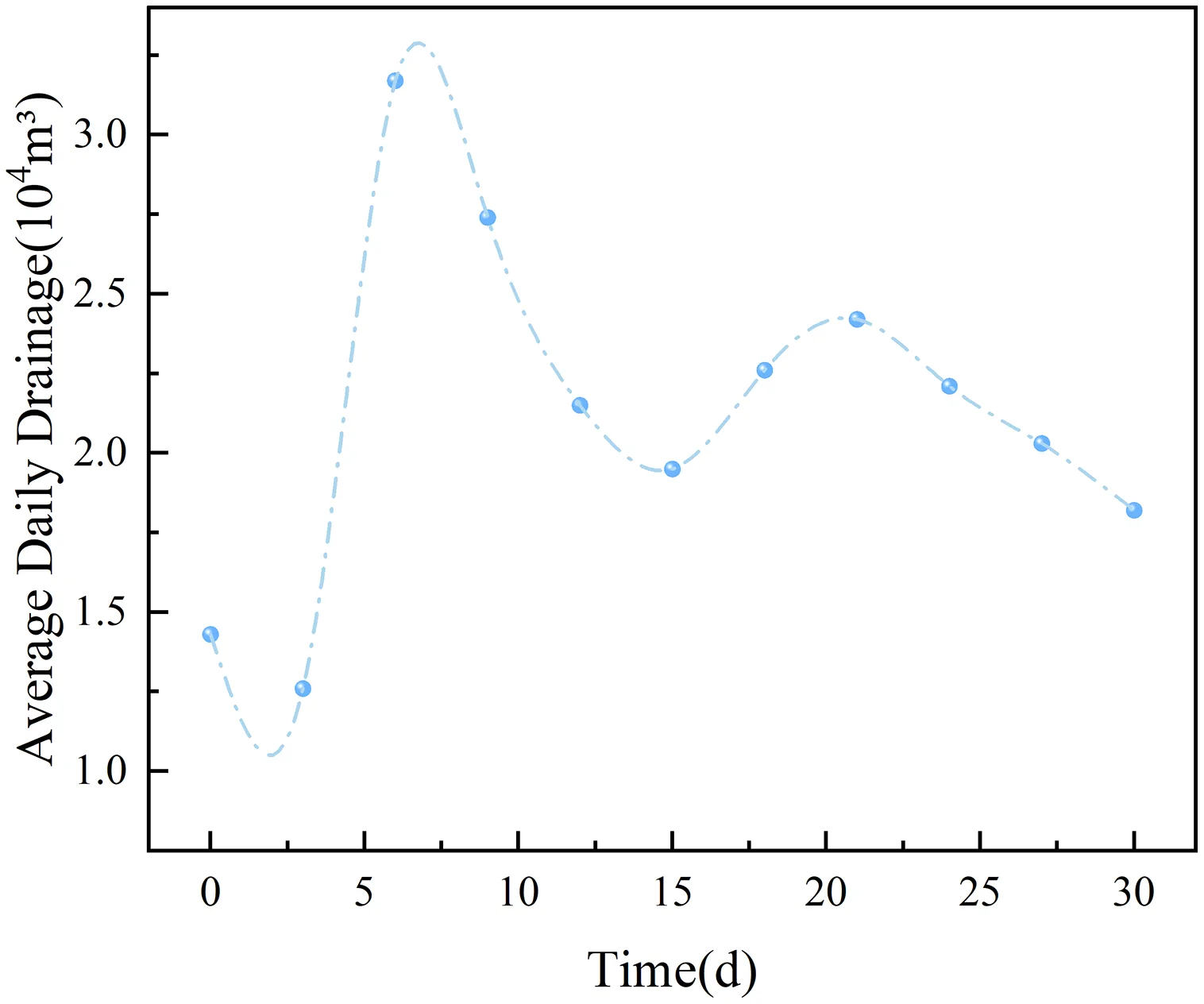
Variation in average daily drainage volume in the field.

**Fig 17:**
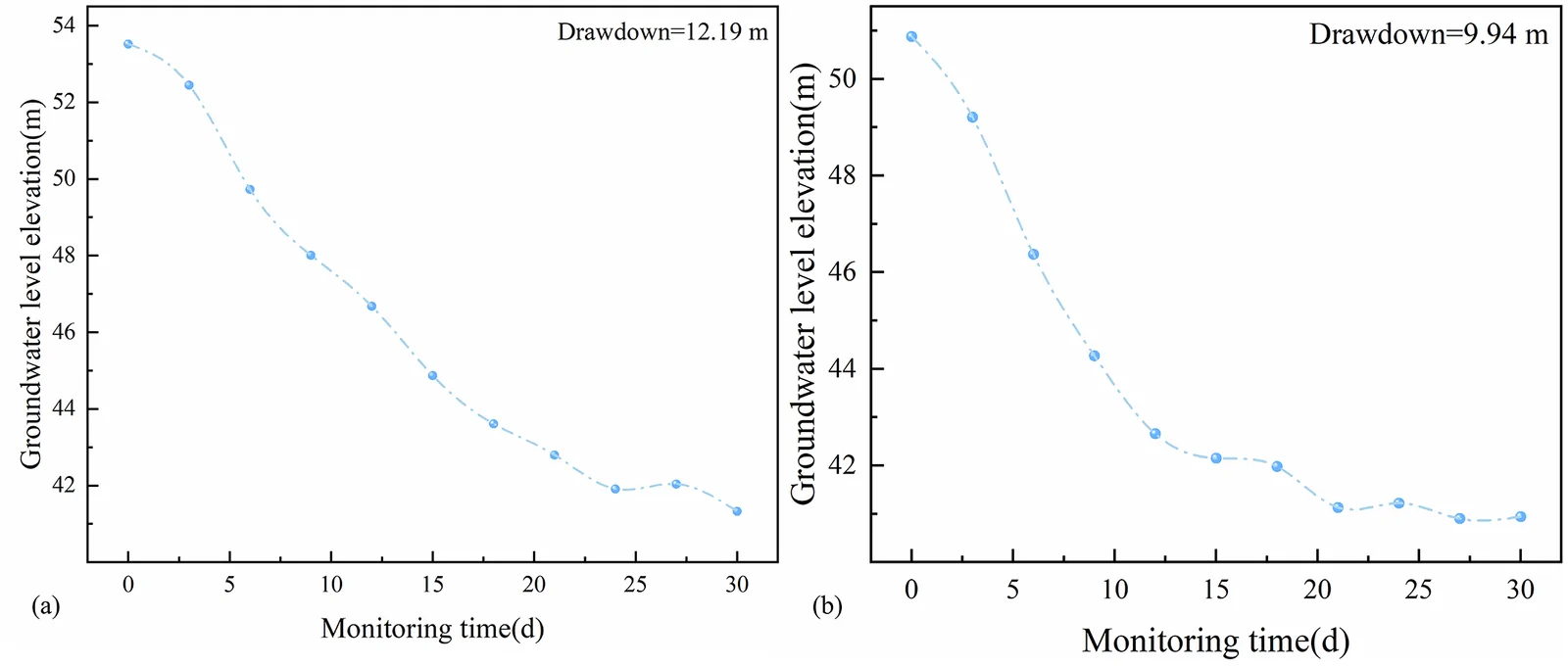
Groundwater-level variation in the aquifers after installation of HDPE perforated drainage pipes. (a) upper aquifer, (b) middle aquifer.

During the one-month monitoring period, the average daily drainage volume was 2.51 × 10^4^ m^3^/day, as shown in Fig 16. The groundwater levels in the upper and middle aquifers decreased by approximately 12.19 m and 9.94 m, as shown in Fig 17, respectively. These measurements provide direct field evidence that the drainage system increased groundwater discharge and produced substantial hydraulic drawdown in the target aquifers.

The numerical model additionally indicated reductions in pore pressure, restriction of saturated-zone expansion, and suppression of continuous plastic-zone development. However, because the field monitoring program did not include dedicated displacement or crack measurements, the agreement between the field and numerical results should be interpreted as hydraulic validation rather than direct deformation validation.

The combined numerical simulation and field validation results indicate that HDPE perforated drainage pipes can effectively improve the internal seepage condition of the internal dump slope, allowing groundwater to be discharged in an orderly manner through the drainage pipes and slope-toe drainage system. This measure helps reduce pore water pressure and limit the expansion of saturated zones within the slope, while also decreasing the risk of slope deformation and plastic-zone development under rainfall conditions. Therefore, the rational arrangement of HDPE perforated drainage pipes can be regarded as an effective engineering measure for improving slope stability during rainfall periods in the internal dump of the Chaoyang open-pit coal mine.

## 5. Discussion

The unconfined compression tests, direct shear tests, and GTS NX numerical simulations revealed the coupled effects of temperature, moisture content, and rainfall on the stability of the internal dump slope in a cold-region open-pit coal mine. The experimental results showed that moisture content and temperature changed not only the stiffness and shear strength parameters of the internal-dump material but also the pore water pressure distribution, deformation response, and safety factor of the slope under rainfall conditions. Overall, increasing moisture content mainly induced mechanical weakening at RT, whereas freezing under subzero temperatures significantly enhanced the stiffness and cohesion of the material, leading to an overall increase in slope safety factor.

### 5.1. Moisture-induced mechanical weakening under room-temperature conditions

Under room-temperature conditions, the mechanical properties of the internal-dump material deteriorated markedly with increasing moisture content. The unconfined compression results showed that the elastic modulus decreased from 1802.4 MPa at 14.0% moisture content to 1003.6 MPa at 26.0%, corresponding to a reduction of approximately 44.3%. The direct shear results showed that cohesion decreased from 18 to 10 kPa, while the internal friction angle decreased progressively from 23.43° to 18.11°. The intermediate internal friction angles were 20.05° and 18.65° at moisture contents of 17.6% and 23.0%, respectively. These results demonstrate that increasing moisture content weakened both the deformation resistance and shear strength of the internal-dump material under unfrozen conditions.

This weakening effect is mainly associated with the influence of water on particle contact conditions and effective stress [22]. Internal-dump materials generally have relatively high porosity and complex particle-size composition. At lower moisture contents, a certain matric suction and structural interlocking can still exist between particles, providing additional apparent cohesion. As moisture content increases, pore water gradually fills the interparticle pores, weakening matric suction and reducing the effective contact force between particles. As a result, the original bonding and interlocking effects are weakened, leading to simultaneous decreases in elastic modulus and shear strength.

At the slope scale, increasing moisture content under room-temperature conditions further reduced the stability reserve of the internal dump slope. The numerical simulation results showed that, at RT, the safety factor before rainfall decreased from 1.405 at 14% moisture content to 1.176 at 26% moisture content, while the safety factor after rainfall decreased from 1.390 to 1.166. This trend is consistent with the decreases in elastic modulus, cohesion, and internal friction angle obtained from the laboratory tests, indicating that the degradation of material strength parameters directly contributes to the reduction in global slope stability [23].

It should be noted that the adverse effect of rainfall on slope stability at RT did not increase monotonically with moisture content. The safety factor reduction rates under the natural moisture content and 23% moisture content were 3.03% and 2.41%, respectively, which were clearly higher than those under the 14% and 26% moisture-content conditions. This indicates that, when the slope is in an intermediate moisture state, a certain matric suction still remains within the material, and rainfall infiltration can significantly reduce the suction contribution and increase local saturation, resulting in a more pronounced decrease in safety factor. In contrast, under high moisture-content conditions, the slope is already in a relatively unfavorable hydraulic state before rainfall, leaving less additional weakening potential for rainfall infiltration.

### 5.2. Freezing-induced strengthening of material strength and slope stability under subzero temperatures

Different from the moisture-induced weakening observed at RT, the internal-dump material exhibited a clear freezing-induced strengthening effect under subzero temperatures. The unconfined compression test results showed that, at the same moisture content, the elastic modulus of the specimens under −5°C and −15°C was much higher than that under RT. For example, at 23% moisture content, the elastic modulus increased from 1200.7 MPa at RT to 2802.3 MPa at −5°C and 3502.1 MPa at −15°C. The direct shear test results also showed that subzero temperatures significantly increased cohesion. For example, at 14% moisture content, cohesion increased from 18 kPa at RT to 53 kPa at −5°C and 61 kPa at −15°C.

This strengthening effect is mainly attributed to ice bonding formed by the freezing of pore water. Under subzero temperatures, part of the pore water undergoes phase transformation and forms ice crystals, which act as bonding and bridging agents between particles. This enhances interparticle connection strength and improves the overall stiffness and shear resistance of the specimen. Freezing affected cohesion and internal friction angle in different ways. Cohesion increased substantially as temperature decreased, indicating that pore-ice bonding provided an additional apparent bonding component between particles. In contrast, the internal friction angle changed only modestly and non-monotonically with temperature. At 14.0% moisture content, the internal friction angle was 23.43° at RT, 21.06° at −5°C, and 21.97° at −15°C. At 23.0% moisture content, the corresponding values were 18.65°, 19.23°, and 19.12°, respectively. Similar small fluctuations occurred at the other moisture contents. Therefore, decreasing temperature did not produce a systematic increase or decrease in the internal friction angle. The strengthening effect of freezing was mainly reflected in the marked increase in apparent cohesion and elastic modulus rather than in a consistent change in frictional resistance.

Temperature and moisture content affected the two components of the Mohr-Coulomb strength envelope differently. Subzero temperature markedly increased cohesion because pore ice and ice bridges provided additional bonding resistance between particles. However, the internal friction angle showed only modest and non-monotonic variations with temperature.

The internal friction angle is mainly associated with particle shape, surface roughness, packing structure, interlocking, and frictional sliding. Cooling can influence these mechanisms through competing processes. The formation of pore ice may restrict particle rearrangement and locally enhance interlocking, which can increase the fitted friction angle. Conversely, ice coatings and unfrozen-water films at particle contacts may reduce direct mineral-to-mineral contact and produce local lubrication, which can decrease the friction angle. The balance between these competing effects depends on moisture content and the spatial distribution of pore water and ice, explaining why no monotonic temperature dependence was observed.

In contrast, increasing moisture content produced a clearer reduction in the internal friction angle. Additional water weakened direct particle contact and interlocking and increased the lubricating effect at contact surfaces. Therefore, moisture content was the dominant factor controlling the friction-angle variation, whereas freezing-induced strengthening was expressed primarily through the increase in apparent cohesion.

The numerical simulation results further demonstrate that freezing-induced strengthening significantly improved the global stability of the slope. At −5°C, the safety factor before rainfall increased from 1.476 at 14% moisture content to 1.535 at 26% moisture content, while the safety factor after rainfall increased from 1.469 to 1.533. At −15°C, the safety factor remained at a higher level, increasing from 1.484 to 1.540 before rainfall and from 1.478 to 1.537 after rainfall. Unlike the decreasing safety factor trend with increasing moisture content at RT, increasing moisture content under subzero temperatures contributed to higher slope stability.

It should be noted that the increase in factor of safety with moisture content under the prescribed subzero-temperature cases cannot be explained by a single mechanical parameter. At both −5°C and −15°C, cohesion and internal friction angle generally decreased as moisture content increased, whereas the elastic modulus increased up to 23.0% moisture content and then decreased moderately at 26.0%. Nevertheless, the calculated factors of safety varied within a relatively narrow range and showed a small overall increase with moisture content. This result reflects the nonlinear response of the slope to the complete set of assigned mechanical parameters and the associated redistribution of stress and deformation, rather than a direct monotonic relationship with cohesion, internal friction angle, or elastic modulus alone. Because the same permeability coefficient was adopted for all temperature cases, the calculated trend should not be attributed to temperature-induced restriction of rainfall infiltration or water migration.

This indicates that temperature changes the role of moisture content in slope stability. At RT, water mainly acts adversely by reducing matric suction and effective stress. Under subzero temperatures, however, frozen water participates in the formation of an ice–soil composite structure, transforming moisture from a weakening factor into a strengthening factor. Nevertheless, this does not mean that higher moisture content is always more beneficial. The unconfined compression test results showed that, under −5°C and −15°C conditions, the elastic modulus reached relatively high values at approximately 23% moisture content and then decreased at 26% moisture content. This suggests that excessive moisture content may lead to uneven pore-ice distribution or reduced structural compatibility, thereby limiting the further development of freezing-induced strengthening.

### 5.3. Coupled temperature–moisture–rainfall mechanism

The results of this study indicate that temperature, moisture content, and rainfall do not independently affect the stability of the internal dump slope as isolated factors. Instead, they jointly influence slope stability by altering the strength parameters of dump materials, the pore water pressure state, and the deformation response of the slope. The unconfined compression and direct shear tests showed that increasing moisture content at RT reduced the elastic modulus, cohesion, and internal friction angle, whereas freezing under subzero temperatures significantly increased specimen stiffness and cohesion. The numerical simulations further showed that the safety factor decreased with increasing moisture content at RT, while it increased with increasing moisture content under −5°C and −15°C conditions. This indicates that temperature changed the role of moisture content in controlling slope stability.

Under room-temperature rainfall conditions, water mainly acts as an unfavorable factor for slope stability. On the one hand, higher moisture content weakens matric suction and effective interparticle contact force, resulting in decreases in elastic modulus, cohesion, and internal friction angle. On the other hand, rainfall infiltration further changes the pore water pressure and saturation distributions within the slope, leading to a reduction in effective stress in localized areas. For internal dump slopes composed of loose dumped materials, the particle-size composition is complex and the pore structure is well developed; therefore, rainfall water can more easily infiltrate through bench platforms, slope-surface cracks, and high-permeability pathways, forming local water-enriched zones. In this case, material strength degradation and pore pressure increase act together, reducing the stability reserve of the slope.

Rainfall sensitivity also showed a nonlinear pattern under different room-temperature moisture-content conditions. The numerical simulation results showed that the safety factor reduction rates under the natural moisture content and 23% moisture content were 3.03% and 2.41%, respectively, which were higher than those under the 14% and 26% moisture-content conditions. This can be explained by the different initial hydraulic states of the slope. Under the low-moisture condition, the slope still has relatively strong matric suction and a higher strength reserve, so short-term rainfall is less likely to cause significant weakening at the global scale. Under the high-moisture condition, the slope is already in an unfavorable hydraulic state before rainfall, leaving relatively limited additional weakening potential. In contrast, under intermediate moisture conditions, the slope retains a certain amount of matric suction while remaining highly sensitive to water migration. Therefore, rainfall-induced suction loss and local saturation effects become more pronounced, leading to larger reductions in safety factor.

Under subzero-temperature conditions, temperature fundamentally changes the role of water. After pore water freezes, ice crystals and ice-bonding structures form, and water no longer acts mainly as a lubricating or softening factor. Instead, it participates in forming an ice–soil composite skeleton. This structure strengthens interparticle bonding, increases specimen stiffness and apparent cohesion, and consequently raises the overall safety factor of the slope. Particularly under higher moisture-content conditions, the amount of freezable water is greater, and the contribution of ice bonding becomes more pronounced. Therefore, the safety factor under subzero temperatures increases with increasing moisture content, which contrasts sharply with the stability reduction caused by increasing moisture content at RT.

The calculated sensitivity to rainfall was lower in the prescribed subzero-temperature cases than in the room-temperature cases. The factor-of-safety reduction rates were 0.13%–0.54% at −5°C and 0.19%–0.40% at −15°C, whereas the maximum reduction at RT reached 3.03%. In the present numerical framework, this difference mainly resulted from the higher elastic modulus and cohesion assigned according to the frozen-specimen test results. These parameters increased the resistance of the slope to deformation and strength reduction during the prescribed rainfall event.

The same permeability coefficient was used for all temperature cases, and the model did not simulate water–ice phase transformation, temperature-dependent hydraulic conductivity, or transient freezing and thawing. Therefore, the lower calculated rainfall sensitivity under subzero conditions should not be interpreted as direct numerical evidence that freezing restricted rainfall infiltration or water migration. In actual frozen slopes, pore-ice formation may reduce hydraulic conductivity and modify infiltration pathways, but this hydraulic effect was outside the scope of the present simulations.

Although the prescribed subzero-temperature cases exhibited higher factors of safety, these results should not be interpreted as evidence of long-term safety throughout seasonal temperature changes. The present simulations did not model transient thawing. Nevertheless, based on the experimentally observed loss of freezing-induced stiffness and cohesion between the subzero and room-temperature states, it can be inferred that the freeze–thaw transition may represent an unfavorable engineering period. As temperature rises, pore-ice bonding weakens or disappears, while meltwater may increase liquid-water content and pore water pressure. If rainfall occurs during this transition, strength reduction and hydraulic deterioration may act simultaneously. This interpretation is an engineering implication supported by the contrast between the prescribed frozen and unfrozen states, rather than a direct simulation of the thawing process.

Therefore, slope stability under coupled temperature–moisture–rainfall conditions has clear stage-dependent characteristics. At RT, increasing moisture content and rainfall infiltration jointly weaken slope stability. During the freezing period, water phase transformation and ice bonding enhance slope strength. During the freeze–thaw transition stage, the disappearance of ice bonding, meltwater recharge, and rainfall infiltration may jointly trigger a rapid decrease in stability. This understanding indicates that stability assessment of internal dump slopes in cold regions should not rely only on the higher safety factor during the freezing period, but should pay particular attention to slope responses under the combined effects of freeze–thaw transition and warm-season rainfall.

### 5.4. Drainage and stabilization mechanism of HDPE perforated drainage pipes

The stabilizing effect of HDPE perforated drainage pipes on the internal dump slope is essentially achieved by modifying the internal seepage boundary conditions and groundwater discharge pathways of the slope. For the internal dump slope of the Chaoyang open-pit coal mine, the upper and middle aquifers are important hydrogeological units controlling the distribution of pore water pressure within the slope. If groundwater within the slope cannot be discharged in time, rainfall infiltration and confined-water recharge may jointly increase local pore water pressure, thereby reducing effective stress and shear strength. Therefore, installing HDPE perforated drainage pipes in the upper and middle aquifers provides preferential drainage pathways for groundwater, transforming the seepage pattern from natural dispersed discharge to concentrated discharge toward the pipe zone.

From the seepage mechanism perspective, the main function of the drainage pipes is to lower the hydraulic head and pore water pressure within the slope. The perforated pipe wall allows groundwater in the aquifers to converge toward the pipe and then be discharged from the slope along the drainage pipe, thereby weakening water-pressure accumulation inside the aquifers. Because pore water pressure is negatively correlated with effective stress, a decrease in pore water pressure increases effective stress and partially restores soil shear strength. This process explains why the high-pore-pressure zone did not expand extensively after rainfall and why the slope maintained a relatively high safety factor after the installation of drainage pipes. The simulation results showed that the safety factor of the slope with HDPE drainage pipes was 1.413 before rainfall and 1.384 after rainfall, both greater than 1.0, indicating that this drainage measure maintained the global stability of the slope under rainfall disturbance.

From the perspective of saturated-zone control, HDPE perforated drainage pipes can weaken local water enrichment after rainfall infiltration. Internal dump materials are generally highly heterogeneous, with complex particle-size distributions and well-developed pore structures. Rainwater can easily infiltrate through bench platforms, slope-surface cracks, and local high-permeability pathways, forming zones of increased saturation in aquifers or low-lying areas. If these high-saturation zones continue to expand, matric suction loss, effective stress reduction, and local shear strength degradation may occur. By enhancing the drainage capacity of the aquifers, the drainage pipes allow water within the slope to converge more rapidly toward the drainage structure, thereby limiting the continuous expansion of saturated zones. This helps reduce rainfall-induced softening in the upper and middle parts of the slope and within the aquifers.

From the perspective of deformation and plastic-zone evolution, the stabilizing effect of drainage and pressure relief is not limited to changes in the hydraulic field but is further transferred to the stress and deformation fields. After pore water pressure decreases, the effective stress within the slope increases, local shear deformation is restrained, and high-displacement zones and plastic strain concentration zones are less likely to connect and form a through-going slip surface. The effective plastic strain results showed that, after installing HDPE perforated drainage pipes, plastic strain was mainly concentrated near local slope-toe areas, the drainage pipes, or geometric transition zones, without forming a continuous plastic deformation band extending from the rear part of the slope to the slope toe. This indicates that although the drainage pipes may cause local stress redistribution around the pipe zone, this local response did not evolve into a failure mode controlling the global stability of the slope.

The field monitoring results further verified the drainage effect of the HDPE perforated drainage pipes. After one month of continuous operation of the drainage system, the groundwater levels in both the upper and middle aquifers showed a continuous decline, with cumulative drawdowns of 12.19 m and 9.94 m, respectively. This indicates that the drainage pipe network effectively enhanced groundwater discharge capacity in the target aquifers and demonstrated at the field scale that the drainage measure could lower groundwater levels and pore water pressure within the slope. These field observations are consistent with the numerical simulation results showing reduced pore pressure, restricted saturated-zone expansion, and restrained deformation concentration, indicating that HDPE perforated drainage pipes have good engineering applicability.

Therefore, the stabilizing effect of HDPE perforated drainage pipes on the internal dump slope can be summarized as a chain mechanism of “drainage and pressure relief–restriction of saturated-zone expansion–suppression of deformation concentration–reduction of through-going slip-surface risk.” First, the drainage pipes provide preferential discharge pathways for groundwater, lowering the hydraulic head and pore water pressure within the aquifers. Second, the reduction in pore pressure limits the expansion of high-saturation zones after rainfall, reducing water-induced softening of the internal dump material. Third, the improved hydraulic condition increases the effective stress of the slope and restrains further development of local displacement and plastic strain. Finally, the likelihood of through-going potential slip surface formation decreases, and the stability reserve of the slope during rainfall is maintained.

It should be noted that the long-term effectiveness of drainage pipes may be constrained by groundwater quality and clogging risk. Field evidence indicates that, when the groundwater contains a relatively high concentration of iron ions, oxidation and precipitation may occur during drainage, forming Fe(OH)_3_ colloids or insoluble Fe_2_O_3_. These precipitates may gradually attach to the drainage pipe and surrounding pores, reducing the effective flow area and hydraulic conductivity. Therefore, in practical engineering applications, HDPE perforated drainage pipes should be not only rationally arranged but also supported by long-term monitoring and maintenance measures, such as periodic groundwater-level observation, drainage-volume monitoring, pipe cleaning, and filter-structure maintenance, to ensure the long-term stable operation of the drainage system.

### 5.5. Engineering implications and limitations

The results of this study provide useful engineering implications for the stability evaluation and control of internal dump slopes in cold-region open-pit coal mines. First, room-temperature and warm-season rainfall conditions should be regarded as critical stages for slope stability assessment. Both the experimental and numerical results indicate that, under unfrozen conditions, increasing moisture content significantly reduces the elastic modulus, cohesion, and internal friction angle of internal-dump materials, further leading to a decrease in slope safety factor. In particular, the slope showed higher sensitivity to rainfall disturbance under the natural moisture content and 23% moisture-content conditions. Therefore, slope safety management in cold-region open-pit coal mines should not focus only on the high-stability state during the freezing period, but should pay particular attention to moisture-content increase, pore-pressure accumulation, and local saturated-zone expansion during the spring freeze–thaw transition period and warm-season heavy rainfall period.

Second, temperature clearly regulates the role of moisture content. At RT, water mainly acts as a weakening factor, whereas under subzero temperatures, pore water freezes and forms ice-bonding structures, causing increasing moisture content within a certain range to improve material stiffness and slope safety factor. This indicates that slope stability in cold regions has strong seasonal dependence. During the freezing period, the slope may exhibit a relatively high stability reserve; however, as temperature rises, the ice-bonding effect disappears, and the frozen water that previously contributed to strength enhancement is transformed into liquid water, which may rapidly increase local pore water pressure and moisture content. Therefore, the freeze–thaw transition period should be regarded as a critical risk window in the long-term stability evaluation of internal dump slopes in cold regions.

Third, drainage and pressure relief are effective engineering measures for improving the rainfall stability of internal dump slopes. The HDPE perforated drainage pipe simulation and field validation results indicate that rationally installing drainage pipes in the upper and middle aquifers can improve groundwater discharge conditions within the slope, reduce pore water pressure, limit the expansion of high-saturation zones, and restrain local deformation and plastic-zone development. Therefore, for internal dump slopes affected by both groundwater and rainfall, an integrated control strategy of “aquifer identification–drainage pathway design–dynamic groundwater-level monitoring–drainage system maintenance” is recommended. The layout of drainage pipes should not be determined solely by slope geometry, but should be comprehensively optimized by considering aquifer positions, groundwater flow direction, slope-toe drainage conditions, and potential slip-zone distribution.

Although this study established an analytical framework integrating laboratory parameter acquisition, GTS NX numerical simulation, and HDPE drainage measure validation, several limitations remain. First, the laboratory tests mainly considered three temperature levels and four moisture-content levels, which can reflect the variation in material mechanical properties under typical hydrothermal conditions, but they did not cover continuous temperature variation, repeated freeze–thaw cycles, or long-term wetting–drying alternation. In actual cold-region internal dump slopes, the materials are subjected to periodic freeze–thaw and rainfall–evaporation alternation, and their structure and strength parameters may undergo cumulative degradation over time. This issue should be further considered in future studies.

Second, the numerical simulation used the representative E–E′ section and a typical rainfall boundary condition, which can reveal the main response patterns of slope stability under temperature, moisture-content, and rainfall effects. However, the model remains a simplification of the complex spatial structure of the actual slope. Internal dump materials are highly heterogeneous, and differences in dumping stage, dumping height, compaction state, and particle-size composition may influence seepage pathways and local deformation patterns. In addition, the rainfall condition adopted in this study was a representative heavy rainfall scenario of 0.05 m/day lasting 72 h, and the slope responses under different combinations of rainfall intensity, rainfall duration, and antecedent moisture condition were not systematically compared. Future studies should further establish transient seepage–stability models under multiple rainfall scenarios.

Third, the simulation and field validation of HDPE perforated drainage pipes mainly focused on the drainage and pressure-relief effect and short-term groundwater-level response, whereas the long-term operational reliability of the drainage system requires further investigation. Groundwater quality may lead to iron-ion oxidation and precipitation, clogging of filter structures, and reduction in drainage capacity, which can affect the long-term drainage efficiency of the pipes. Therefore, future studies should incorporate long-term field monitoring to systematically evaluate drainage-volume attenuation, groundwater-level recovery, pipe clogging, cleaning and maintenance cycles, and optimized drainage pipe layout, thereby improving the long-term reliability of engineering control measures.

Overall, the experimental–numerical–engineering validation framework proposed in this study can provide a reference for the stability evaluation of internal dump slopes in cold regions. For similar cold-region open-pit coal mine projects, temperature, moisture content, rainfall infiltration, and groundwater discharge conditions should be comprehensively analyzed as coupled factors. In slope control, priority should be given to warm-season rainfall, freeze–thaw transition, and aquifer drainage conditions. Only by integrating material strength variation, hydrogeological response, and engineering drainage measures can the stability evolution of internal dump slopes during long-term service be more accurately evaluated.

## 6. Conclusion

This study investigated the mechanical behavior and slope-scale stability response of the internal dump at the Chaoyang open-pit coal mine under prescribed temperature, moisture-content, rainfall, and drainage conditions. The main conclusions are as follows:

(1) Temperature and moisture content jointly controlled the stiffness and shear strength of the internal-dump material. At room temperature, the elastic modulus decreased from 1802.4 MPa at 14.0% moisture content to 1003.6 MPa at 26.0%, corresponding to a reduction of 44.3%, while cohesion decreased from 18 to 10 kPa. Under −5°C and −15°C conditions, both elastic modulus and cohesion increased markedly and generally reached relatively high values at intermediate moisture contents. The internal friction angle varied modestly and non-monotonically with temperature, decreasing from 23.43° to 18.11° with increasing moisture content at room temperature. Therefore, the strengthening associated with subzero temperatures was mainly reflected in increased apparent cohesion and stiffness rather than a systematic increase in internal friction angle.
(2) Temperature changed the role of moisture in both material behavior and slope stability. Under unfrozen conditions, additional water reduced matric suction, weakened particle contacts, and decreased the factor of safety. The pre-rainfall factor of safety at room temperature decreased from 1.405 to 1.176 as moisture content increased from 14.0% to 26.0%. Under subzero conditions, pore-ice bonding restricted particle movement and increased cohesion, causing the factor of safety to increase with moisture content within the investigated range. At −15°C, the post-rainfall factor of safety increased from 1.478 to 1.537. These results indicate that the spring thawing period and warm-season moisture increase are more critical than the fully frozen period because the ice-bonding contribution may disappear while liquid-water content and pore pressure increase.
(3) Rainfall altered the hydraulic and deformation fields but did not cause global instability under the simulated conditions. For the representative 14.0% moisture-content condition at room temperature, 72 h of rainfall reduced the factor of safety from 1.405 to 1.390. The maximum total displacement was approximately 3.46 × 10^−2^ m, horizontal displacement remained at the millimeter scale, and the maximum shear strain was approximately 8.98 × 10^−4^. No continuous shear band or through-going potential slip surface developed. Across all room-temperature cases, the rainfall-induced reduction in factor of safety ranged from 0.85% to 3.03%, whereas all reductions under subzero conditions were below 0.60%. The lower calculated rainfall sensitivity of the subzero cases mainly resulted from their higher assigned elastic modulus and cohesion; temperature-dependent permeability and water–ice phase transformation were not included in the present model.
(4) HDPE perforated drainage pipes effectively improved the hydraulic condition and stability reserve of the slope. Under the room-temperature natural-moisture condition, drainage increased the factors of safety before and after rainfall from 1.221 and 1.184 to 1.413 and 1.384, corresponding to increases of 15.72% and 16.89%, respectively. Drainage restricted the expansion of high-pore-pressure and high-saturation zones and prevented local displacement and plastic-strain concentrations from developing into a continuous failure zone. Field monitoring recorded an average drainage discharge of 2.51 × 10^4^ m^3^/day and groundwater-level drawdowns of 12.19 and 9.94 m in the upper and middle aquifers, respectively. These field observations provide hydraulic validation of the drainage effect, although long-term structural deformation monitoring and drainage-performance assessment remain necessary.

## Supporting information

S1 File Direct shear test data English. (XLSX)

S2 File Unconfined compression test data English. (XLSX)
